# Catalysts Design and Atomistic Reaction Modulation by Atomic Layer Deposition for Energy Conversion and Storage Applications

**DOI:** 10.1002/EXP.20240010

**Published:** 2025-04-21

**Authors:** Myung‐Jin Jung, Alireza Razazzadeh, Hasmat Khan, Se‐Hun Kwon

**Affiliations:** ^1^ School of Materials Science and Engineering Pusan National University Busan Republic of Korea; ^2^ Institute of Materials Technology Pusan National University Busan Republic of Korea

**Keywords:** atomic layer deposition, electrocatalysis, energy conversion and storage, heterogeneous catalysts, photoelectrocatalysis

## Abstract

Atomic layer deposition (ALD) technique has emerged as a fascinating tool for the design and synthesis of heterogeneous catalysts with atomic precision for energy conversion, generation, and storage applications. Here, we demonstrate the importance of the ALD for catalyst design by citing recently reported works, in particular, the emphasis has been given to the surface/interface engineering of catalysts for improving their catalytic efficiency in energy applications. To get insight into the reaction mechanism, the ALD‐based routes for catalyst synthesis may revolutionize the field of sustainable energy conversion and storage. Moreover, the synthesis of supported nanoparticles with controlled shape and size has attracted great attention in catalysis owing to their unique properties. By taking advantage of the ALD, it is possible to synthesize catalysts at the atomic scale, particularly, site‐selective ALD provides tremendous opportunities in catalytic efficiency and selectivity studies. Moreover, this review illustrates diverse heterogeneous catalysts with their limitations for energy‐related applications and how the ALD technique can facilitate overcoming them. Finally, we deliberate the advancement in the ALD technique on heterogeneous catalyst design, and interface engineering of catalysts, and outline future perspectives of this technology in catalysis.

## Graphical abstract

This review demonstrates the development of heterogeneous catalysts for energy conversion and storage applications using the atomic layer deposition (ALD) technique. A comprehensive survey of various types of energy‐related catalysts has been conducted. Moreover, this review highlights the advancements of the ALD technique in synthesizing efficient catalysts for energy applications.

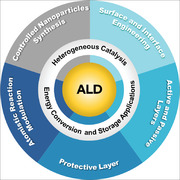

## 1 Introduction

Increased worldwide population, rapid urbanization, and industrial revolution are inextricably linked with the huge energy consumption [1]. Until now, around 90% of the world's total energy demand is fulfilled by conventional fossil‐based fuels [1]. The huge utilization of fossil fuels releases greenhouse gases that cause catastrophic climate change and other environmental issues. Therefore, finding viable and sustainable energy resources as alternatives to conventional fossil fuels is of utmost importance. In this regard, heterogeneous catalysis that requires distinct phases for the catalyst and the reactants, particularly electrocatalysis and photoelectrocatalysis has recently attracted significant attention due to their eco‐friendliness and generation of green energy [1, 2, 3, 4, 5]. Catalysts play an important role in electrocatalytic/photoelectrocatalytic energy conversion and storage applications. And, the heterogeneous catalysts are advantageous compared to the homogeneous catalysts due to their robustness and easy separation from the reaction mixtures [6]. However, the heterogeneity and complexity of heterogeneous catalysts’ atomic and chemical structures are major concerns for catalytic efficiency evaluation. For example, a metal nanoparticle (NP) is covered by several atoms that have different edges, faces, and coordination environments [7]. Moreover, the presence of defects such as vacancies, kinks, atomic steps, stacking faults, and twin boundaries in such catalysts makes the catalytic process even more complex. In addition, precise determination of the “active center” of a heterogeneous catalyst is a formidable task, and hard to understand the catalytic mechanism at the atomic scale [2, 8]. The worst thing is that the impregnation strategy is used to prepare the industrial catalysts, in which a metal salt precursor is deposited on a support followed by pyrolysis at an elevated temperature under a suitable gaseous environment. The catalysts prepared using this strategy are broadly distributed, with different shapes and sizes of catalyst NPs, inhomogeneous surface structure, and poor interaction with the support [6, 8, 9].

Metal catalysts on support are used in the diverse fields of heterogeneous catalysis reactions, including electrocatalytic reactions, photocatalysis, automobile exhaust treatment, biomass conversions, chemical synthesis, and so on [10]. As the size of catalyst NPs is intimately related to their catalytic efficiency, developing a precisely controlled synthetic method to reduce the catalyst's size is highly desirable. The recent progress in conventional wet‐chemical methods including ion exchange, wet‐impregnation, deposition‐precipitation, and co‐precipitation for catalyst synthesis facilitates decreasing metal loadings and choosing suitable supports to suppress aggregations of atoms to deposit sub‐nanometer clusters of catalysts on supports [8, 11]. Besides the wet‐chemical methods, host–guest strategy, chemical reduction, and strong electrostatic adsorption methods have also been established to control the size of the clusters [12]. However, the reduction in the size of NPs and controlling the number of metal atoms in the nanoclusters by the above‐mentioned methods are generally elusive due to hard control of the aggregation process at the atomic scale, resulting in average catalytic performance and obscuring insight into the structure‐activity relationships of the catalysts [13].

On the other hand, the catalytic performance not only depends on the size of the heterogeneous catalysts but is also dependent on their interfacial properties [14]. Interface engineering such as nano‐alloying, heteroatom doping, and heterojunction formation of the catalysts facilitate improving catalytic efficiency [15]. Specifically, supported metal particles where an oxide material is used as a support are important materials in heterogeneous electrocatalysis. When metal NPs interact with the oxide surface then they form metal/oxide interfaces that greatly influence the surface morphology and electronic structure of the metal particles, tuning the catalytic efficiency of the catalysts [16]. As the interface engineering of catalysts requires atomic‐level precision, it is challenging to precisely modify the metal/oxide interfaces with conventional methods such as sequential deposition, impregnation, co‐precipitation, the sol–gel method, annealing treatment, and gas‐induced surface segregation [16, 17]. Moreover, the catalysts (metal NPs on support) prepared by conventional methods are not structurally uniform, and thus, the understanding of metal/oxide interface structures is unsatisfactory. Recently, some studies on combined theoretical calculations and in‐situ or operando spectroscopy techniques have revealed that the metal/oxide interface plays an important role in catalytic performance. Typically, metal/oxide interface structures have unique and complicated electronic states, interactions, and boundary structures that arise from polarization, hybridization, and charge transfer [18]. Therefore, a small modification in the interface causes a significant difference in the material properties of the catalysts, leading to a difference in catalytic performance. Several factors such as composition, particle size, electronic state of interface elements, and microstructures interfere with the catalytic activity [19]. Thus, it is of utmost importance to develop new methods to precisely control the metal/oxide interface structures and mechanistic insight into the interface catalysis.

**FIGURE 1:**
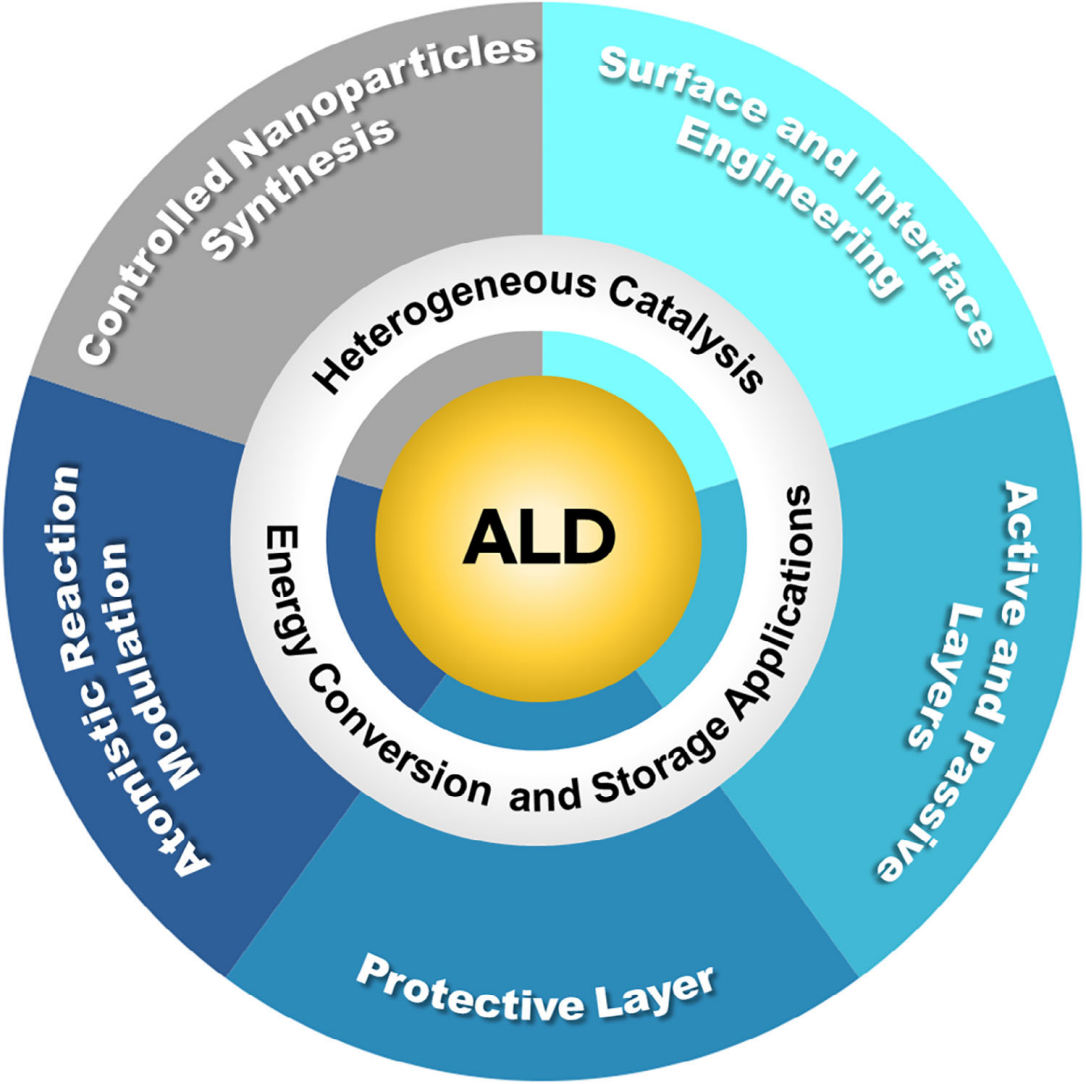
A schematic drawing represents the main content of the review.

In the past few years, the atomic layer deposition (ALD) technique has become popular in the field of heterogeneous catalysis owing to its precise and reproducible deposition abilities [20]. A total number of 1794 articles has been published on ALD to synthesize catalysts, of which the majority of the articles (1393) have been published in the last decade as per the “Web of Science” database on 20th May 2024 with the keywords “atomic layer deposition” and “catalysis”, implying the recent involvement of the ALD technique on the preparation of the advanced catalysts. More comprehensive review articles on the ALD technique are required to design efficient catalysts and tune their atomistic reaction for various energy conversion and storage applications. ALD technique is a sophisticated film growth and NP deposition technology [20]. In the ALD technique, a self‐limiting layer‐by‐layer chemical reaction occurs between gaseous metal precursors and the surface of a solid substrate, resulting in the deposition of controllable films or NPs [21]. The ALD provides a diverse range of materials deposition including metals, metal oxides, nitrides, sulfides, polymers, and inorganic–organic hybrid films and the self‐limiting nature of ALD facilitates to precisely control of the deposited film thickness or size of NPs with excellent conformity, reproducibility, and uniformity [21, 22]. In modern technology, the ALD technique has attracted considerable attention for the precise design and synthesis of advanced catalysts with high performance. Taking advantage of the ALD technique, it is possible to develop uniform metal/oxide interfaces with isolated and size‐controllable metal particles, alloys, and single atoms on oxide supports or other carbon‐based supports. The main content of this review is shown in Figure 1.

The unique features of ALD make it particularly beneficial for designing energy‐related catalysts, where uniformity, precision, and tailored properties are crucial. However, high‐cost, slow deposition rate, and limited materials deposition make the ALD technique incapable of versatile materials deposition and large‐scale fabrication [21, 22]. Therefore, scalability of ALD technique is required for industrial catalysts production and retaining structural and catalytic integrity in corrosive media and high temperature. Generally, catalysts used in electrochemical energy conversion and storage applications such as electrolyzers and fuel cells face challenges from NPs agglomeration, dissolution during cycling, or sintering. In this regard, appropriate support design or coating to anchor catalysts NPs and prevent them from agglomeration or migration are required. It is worth mentioning that ALD technique can effectively form core–shell structures with tailored coating thickness and catalyst loading on support materials. Although some reports are available on interfacial layer deposition via ALD technique in between support and catalyst with strong catalyst‐support interactions that prevents agglomeration and migration during electrochemical operations [21, 22], unmet needs in scalability of ALD technique such as conformal coating on three‐dimensional complex geometries and large‐scale catalysts synthesis are to be considered for industrial application of this technique in energy field. Additionally, other scalability issues of ALD technique such as energy‐efficient ALD techniques and compatibility of ALD equipment with conventional catalysts synthesis methods for enabling hybrid deposition to be approached by low‐temperature or plasma‐enhanced ALD (PEALD) for sustainable production and developing modular ALD systems that can be integrated into conventional fabrication methods for large‐scale catalysts production. It is noted that regulation of catalyst properties at the atomic scale requires materials design with specific alternation of chemical, physical, and electronic properties to maximize catalytic activity, stability, and selectivity. Although, recent advancements in ALD techniques and in‐situ/operando characterization have significantly allowed us to manipulate catalysts at atomic‐scale, some major challenges still remain on achieving precise atomic arrangements to generate specific active sites in complex or multicomponent catalysts, surface and interface phenomena (e.g. catalyst‐support interaction and the interaction between adsorbed species), obtaining uniformity in structure, composition, and electronic properties over a catalyst's surface, and atomic‐scale tuning of multicomponent catalysts (e.g. alloys, high‐entropy oxides, and hybrid materials).

In this review, we emphasize the recent advancement of catalyst design and their atomic‐scale reaction modulation for energy conversion and storage applications by the ALD technique. For this, we first discuss the versatility and uniqueness of the ALD technique for size‐controlled catalyst synthesis and catalysts/support interface engineering. Second, we demonstrate the critical aspects of heterogeneous catalysts for energy conversion, generation, and storage applications and review the important role of ALD technology in achieving them. Third, an overview of the ALD technique has been provided for designing different catalysts for electrochemical energy conversion, fuel cells, solar cells, photocatalysis, photoelectrochemical (PEC) water‐splitting, photoreforming, battery systems, and supercapacitor applications. Then, the advantages of different ALD techniques are evaluated to provide insight into the catalytic mechanism of the different applications. This review illustrates diverse heterogeneous catalysts with their limitations for energy‐related applications and how the ALD technique can facilitate overcoming them. Finally, we deliberate the advancement in the ALD technique on heterogeneous catalysts design and their interface engineering and outline future perspectives of this technology in catalysis.

## 2 Basic Principle and Advantages of ALD Technique

ALD is a versatile vapor phase deposition technique that enables the production of highly specialized thin films on a wide range of substrates [23]. ALD is a technique at the atomic scale through self‐limiting chemisorbed reaction on a substrate by injecting gas phase precursor and reactant in sequential pulse.

In general, ALD technique involves four distinct steps. Once the substrate is loaded into the ALD chamber and the chamber is purged, the precursors are injected into the reaction chamber for a set pulse time and the molecules then react with the functional groups of substrates sufficiently. Then the chamber is purged with inert gas to remove any unreacted initial precursor molecules and reaction byproducts. Then reactant is also injected into the reaction chamber for a set pulse time. Reactant molecules used in the ALD technique can possess varying oxidation strengths, such as H_2_O, O_2_, O_3_, and O_2_ plasma, as well as reducing agents like H_2_ and NH_3_ plasma. Reactant molecules can play the role of effective precursor ligand remover, and finally, a monolayer of surface species would be assembled. Then the chamber is purged again with inert gas to remove any unreacted reactant molecules and reaction byproducts. These four steps constitute an ALD growth 1 cycle together and this is a very precise technique [24].

**FIGURE 2:**
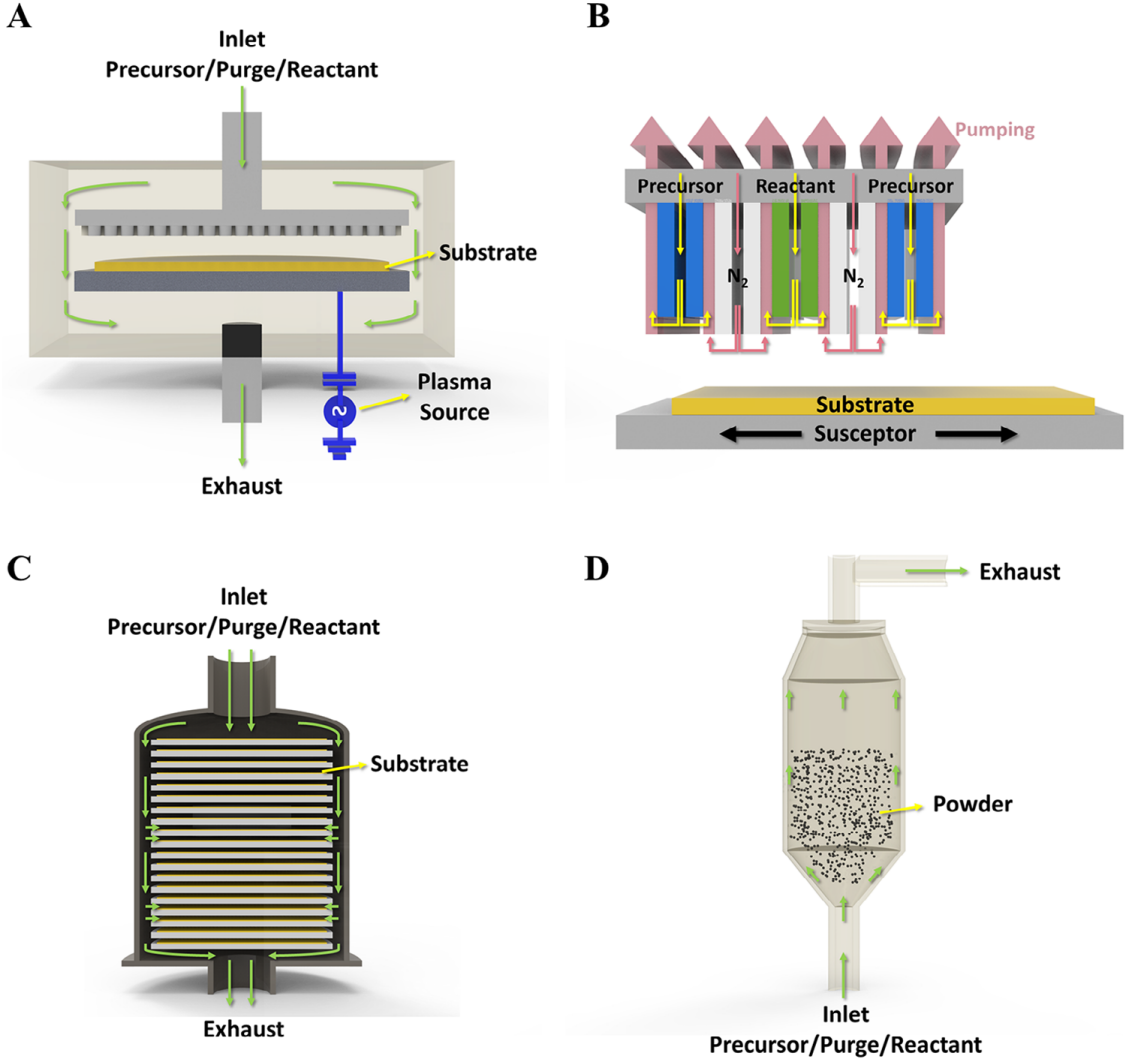
Schematic of the reactors of some advanced ALD equipment: (A) PEALD with showerhead type reactor, (B) SALD, (C) batch‐ALD, and (D) FBR‐ALD.

By utilizing the above‐mentioned steps and self‐limiting surface reactions of ALD technology, which provides functional properties through angstrom‐level precise control of thickness and composition by the number of ALD cycles, excellent uniformity and conformal coverage on substrates with high aspects ratio features, such as over‐complex three‐dimensional (3D) structures, nanowires, and complex pore structures. Especially, in catalysis sub‐monolayers, single sites or NPs could adjust and achieve the number of NPs and sizes by the number of ALD cycles [25]. With these advantages, ALD is emerging as a powerful technology for many industrial and research applications, especially nanomaterials and next‐generation semiconductor processes [23, 26, 27, 28, 29, 30, 31]. Moreover, various types of advanced ALD techniques have recently been developed depending on the reaction activation energy. The advanced variants of ALD can be classified as energy‐ALD technique where external energy sources like thermal energy, plasma, electric potential are used to enhance the chemical reactions during the deposition process (e.g. thermal‐ALD, PEALD, and electric potential‐assisted ALD), high throughput ALD (e.g. spatial ALD, batch‐ALD, and roll‐to‐roll ALD), ALD techniques for powder coating (e.g. fluidized bed reactor‐ALD, rotating drum‐ALD, and spouted bed‐ALD), and ALD for polymeric and hybrid materials, including organic‐inorganic composites (e.g. molecular layer deposition and template‐assisted ALD). These alternative techniques expand the versatility of ALD for diverse materials deposition and industrial requirements. Among them, thermal‐ALD, PEALD, electric potential‐assisted ALD (EA‐ALD), batch‐ALD, spatial ALD (SALD), fluidized bed reactor‐ALD (FBR‐ALD), and molecular layer deposition (MLD) have been widely used for materials deposition on various substrates. Reactors of some advanced ALD equipment have been schematically illustrated in Figure 2. For instance, thermal‐ALD operates as a surface‐driven process where layer growth occurs exclusively through surface reactions. This enables excellent thickness control and conformality, regardless of the substrate geometry or reactor design. It typically requires relatively high process temperatures (150–350°C), which can pose challenges when processing thermally sensitive substrates and precursors [23]. In such cases, PEALD is often utilized to overcome these temperature limitations.

PEALD utilizes plasma excitation during the reactant exposure step to generate highly reactive species such as electrons, ions, and radicals (Figure 2A). The key advantage of PEALD is its ability to achieve high‐quality thin films at significantly lower process temperatures compared to that of the thermal‐ALD. The high reactivity of plasma species enables the comparatively fast deposition with high fidelity films that are difficult to achieve using the conventional thermal‐ALD. PEALD also provide numerous advantages, including compatibility with a wide variety of precursors and reactants. Additionally, its process versatility enables deposition on a diverse range of substrates, making it suitable for applications across various fields. However, in PEALD, the interaction of energetic plasma species (e.g. ions, radicals) with the substrate can lead to damage, which may degrade device performance. Furthermore, in high aspect ratio structures (e.g. deep holes or trenches), radicals undergo multiple wall collisions, leading to surface recombination and lose reactivity before reaching the bottom of the structure, resulting in decrease conformality in complex 3D structures [23]. Furthermore, EA‐ALD has recently emerged as a technique to enhance the uniformity and quality of ultrathin films. This approach applies an electric potential to the substrate, generating an electric field that enhances the adsorption of precursor molecules and regulates the ALD technique by modifying the bonding strength of surface functional groups. The applied electric field increases the collision and adsorption rates of precursor molecules, resulting in higher nucleation density during the initial ALD step. Tuning the applied electric potential enables precise control over surface reaction kinetics and nucleation behavior. As a result, EA‐ALD can improve the microstructure and chemical composition of the deposited films, leading to highly uniform and high‐quality ultrathin films [31].

SALD was also developed to increase the throughput. Unlike conventional ALD, which relies on time‐resolved pulsing and purging of precursors and reactants, SALD employs spatially separated precursor, reactant zones (Figure 2B). In this approach, the substrate is continuously exposed to precursor and reactant gases as it moves between physically separated zones, where each half‐reaction occurs. In SALD, when the substrate is allowed to access the first half‐reaction zone for a sufficient duration, a saturated monolayer form from a specific precursor. Subsequently, the substrate is moved to the second zone for the subsequent half‐reaction to complete the ALD cycle. Alternatively, gas supplies can move while the substrate remains fixed. The use of SALD eliminates the need for purge steps between the injection of the precursor and the reactant and the deposition rate in SALD is not limited by individual cycle times but is determined by the time required for the substrate to transition between reaction zones, making it suitable for large‐scale production [26].

Additionally, batch‐ALD is designed to enhance throughput by simultaneously processing multiple substrates in a single deposition cycle. Unlike single‐wafer ALD systems, which allow one wafer or substrate at a time, batch‐ALD allows multiple substrates to be placed within a chamber and enabling the simultaneous deposition of thin films on several substrates (Figure 2C). Batch‐ALD ensures excellent uniformity of film thickness across all substrates due to its self‐limiting surface reactions, a hallmark of the ALD technique. By processing multiple substrates in parallel, batch‐ALD significantly increases productivity, making it ideal for industrial‐scale applications of thin film deposition for solar cell layers and transparent conductive oxides [31].

Another specialized ALD technique designed for the coating of fine powders and particles is an FBR‐ALD. In this technique, a fluidized bed reactor (Figure 2D) is used to achieve uniform distribution of precursor gases over the surface of individual particles. The fluidized state of the particles allows the precursors to spread all surfaces uniformly, ensuring consistent film thickness and quality across the particles that results conformal and precise deposition of thin films on the high‐surface‐area materials. The FBR‐ALD facilitates atomic‐level control that results in highly conformal coatings even on complex and porous structures. Moreover, it can process large quantities of particles in a single batch, making this technique suitable for industrial applications [26]. Moreover, MLD is an advanced variant of ALD, extends the methodology to deposit organic, polymeric, or hybrid organic‐inorganic composite films with atomic or molecular‐level precision. It is particularly useful for creating functional, flexible, and hybrid materials for a wide range of applications such as energy storage devices and membranes. MLD utilizes organic precursors containing functional groups that react with the substrate. Moreover, alternating organic and inorganic precursors pulses in MLD allow the fabrication of hybrid films combining properties of both types of materials. MLD can also be operated at lower temperatures than the conventional thermal‐ALD, making this technique suitable for heat‐sensitive substrates [2].

In recent years, ALD received huge attention in different energy‐related fields such as battery and catalysis due to the aforementioned beneficial characteristics [25]. Table 1 summarizes the widely used variants of ALD techniques with various precursor materials, reactants, reaction conditions, and types of deposited‐materials.

**TABLE 1:** Summarizes the variants of ALD techniques with various precursor materials, reactants, reaction conditions, and types of deposited‐materials [23, 25, 26, 31].

ALD	Precursors	Reactants	Deposition conditions	Materials
Thermal ALD	Metal halides (e.g. TiCl_4_, SnCl_4_, WF_6_, HfCl_4_, VOCl_3_ etc.), metal alkoxides (e.g. Al(OEt)_3_, Ti(OEt)_4_, Ni(dmamp)_2_ etc.), organometallics (e.g. Cp_2_Ni, (EtCp)_2_Ru, (Me_5_Cp)_2_Sr, (iPrCp)_3_La, Cp_2_MeZr, (MeCp)Pt (Me)_3_ etc.), and metal‐organic compounds (e.g. Pt(acac)_2_, Al(NMe_2_)_3_, Ni(acac)_2_ etc.)	H_2_O vapor, O_2_, O_3_, NO_2_, H_2_O_2_, NH_3_, N_2_H_4_, H_2_S, sulfur vapor, acetylene (C_2_H_2_) gas, hydrogen selenide (H_2_Se) gas, phosphine gas (PH_3_) etc.	Typical temperature range: 150–350°C	Metal/metal oxides/nitrides/sulfides/carbides/selenides/phosphides
PEALD	Metal alkoxides, organometallics, and metal‐organic compounds	Plasma‐activated N_2_, H_2_, O_2_, NH_3_, H_2_S, C_2_H_2_, H_2_Se, PH_3_ etc.	Plasma‐assisted ions and radicals under lower temperature range compared to thermal ALD	Metal/metal oxides/nitrides/sulfides/carbides/selenides/phosphides
EA‐ALD	Metal halides, metal alkoxides, organometallics, and metal‐organic compounds	H_2_O vapor, O_2_, O_3_, oxygen plasma, NH_3_, N_2_‐plasma, N_2_H_4_ etc.	Applied electric potential range from a few volts to hundreds of volts, depending on the system	Metal/metal oxides and metal nitrides
SALD	Metal halides, metal alkoxides, organometallics, and metal‐organic compounds	H_2_O vapor, O_2_, O_3_, NO_2_, H_2_O_2_, NH_3_, N_2_H_4_, H_2_S, sulfur vapor, C_2_H_2_, H_2_Se, PH_3_ etc.	Lower temperature range than thermal ALD with fast deposition rate	Metal/metal oxides/nitrides/sulfides/carbides/selenides/phosphides
FBR‐ALD	Metal halides, metal alkoxides, organometallics, and metal‐organic compounds	H_2_O vapor, O_2_, O_3_, NO_2_, H_2_O_2_, NH_3_, N_2_H_4_, H_2_S, sulfur vapor, C_2_H_2_, H_2_Se, PH_3_ etc.	Low temperature range (50–150°C), moderate‐temperature range (150–300°C), and high‐temperature range (300–600°C)	Metal/metal oxides/nitrides/sulfides/carbides/selenides/phosphides
MLD	Mainly metal‐organic compounds, metal alkoxides, and organometallics	Ethylene glycol, glycerol, ethylenedioxythiophene etc.	Lower temperature range than thermal ALD	Organic–inorganic hybrid films and organic polymers

### 2.1 Versatile Material Deposition

Recent advancement in the ALD technique allows for the deposition of a wide variety of materials. In general, the following groups of materials can be synthesized by ALD: oxides, metals, nitrides, and sulfides, and many related ALD techniques have been researched and developed [20, 21].

**FIGURE 3:**
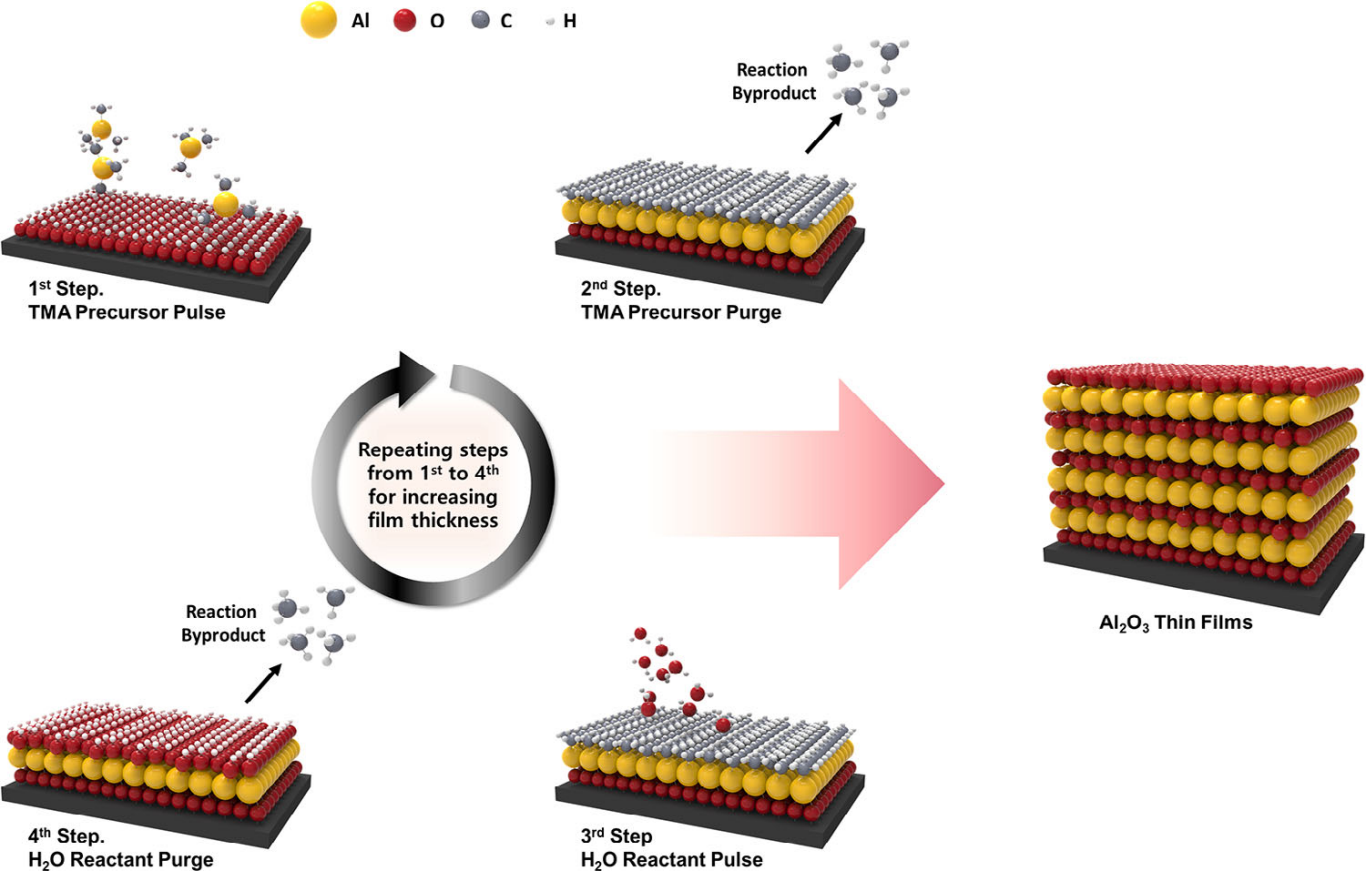
Schematic shows the self‐limiting growth of ALD‐Al_2_O_3_ film by using TMA as precursor for aluminum and H_2_O as reactant. The schematic represents 4 consecutive steps, 1st step: TMA precursor pulse on reactive substrate, 2nd step: precursor purge to remove reaction byproducts, 3rd step: H_2_O reactant pulse, and 4th step: reactant purging to eliminate byproducts. Desired film thickness can be obtained by repeating the consecutive steps from 1st to 4th steps.

Firstly, oxide materials have been most extensively studied using ALD. To form oxides using ALD, it needs to utilize a highly reactive gas that can be supplied with oxygen. Therefore, in the case of oxide materials, H_2_O is mostly used as a reactant, and other types of reactants such as H_2_O_2_, O_2_, O_3_, O_2_ radical, NO_2_ etc. are also used. In the ALD technique, after a specific precursor is injected and a purge process with inert gas is performed, subsequently the reactant is injected, and hydroxyl functional groups on the surface of the substrate facilitate the ligand exchange reaction, which acts as a mediator of the reaction occurring on the surface. After the injection of the reactant, the purge step is further performed to remove byproducts and residual reactant, the process is repeated to achieve the desired film thickness. The deposition of Al_2_O_3_ thin films by the ALD, which are the most common oxide material, can be illustrated as an example (Figure 3). Using trimethyl aluminum (Al(CH_3_)_3_, TMA) as a precursor and H_2_O as a reactant as an example (Equations 1 and 2), when TMA precursor is injected into the OH‐terminated surface, CH_4_ byproducts are generated by reacting with functional groups on the substrate surface, resulting in an Al─O─Al(CH_3_)_2_ surface. After removing the byproducts through a purge, H_2_O reactant is injected, and the H_2_O reacts with the Al(CH_3_)_2_ functional groups on the surface to produce CH_4_ byproducts, resulting in an Al‐OH surface. The purge process also proceeded. This reaction process is repeated alternately to grow Al_2_O_3_ thin films, and similar thin film growth behavior is also observed for other metal oxide materials.

(1)

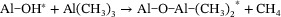



(2)

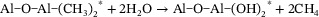




The oxide thin films thus formed mostly exhibit Frank‐van der Merwe growth behavior. Frank‐van der Merwe's growth behavior is also known as bi‐dimensional growth or layer‐by‐layer growth. In this growth behavior, the surface adhesive force is stronger than the intra‐atomic cohesive energy, resulting in layer‐by‐layer growth on the substrate, i.e. the atoms completely cover the surface, creating a complete monolayer before the next layer is formed on top [32].

Besides metal oxides, a lot of research has recently been performed on depositing metal by using ALD. In the case of metals, it is known that metal thin films can be deposited by ligand combustion reaction and reduction reaction utilizing plasma. In the case of noble metals (Ru, Rh, Pd, Os, Ir, Pt), oxidizing agents such as O_2_, O_2_ plasma, O_3_, and in some cases, NH_3_ plasma are used as reactants [20, 21, 25, 26, 27, 28, 29, 30, 31]. In the ALD technique, the ligands of the metal precursor react with the reactant and burn off, resulting in the deposition of pure noble metal. H_2_ plasma has also been widely used as a reactant for metal‐ALD. This is because, through the reaction with H_2_ radicals through H_2_ plasma, the metal‐ligand can be removed as CH_4_ gas if it is composed of carbon, or H_2_O gas if it is composed of oxide.

In the case of the thermal metal‐ALD technique, in the initial stages of film growth in actual ALD techniques, a phenomenon of gradual changes in the amount of adsorbed precursor and reactant is inevitably encountered until the substrate surface transforms into a fully complete film surface. Particularly, when the adsorption on the substrate surface is lower than that on the film surface, it leads to a high incubation period and initial nucleation delay. In addition, when there are a sufficient number of chemically active species present on the substrate, films grown by ALD typically follow the Frank van der Merwe growth mechanism, resulting in layer‐by‐layer film growth. However, when conducting metal‐ALD techniques on substrates with relatively lower surface energy, the substantial difference in surface energy between the substrate and the metal leads to the initial formation of metal NPs oriented towards minimizing surface area, thereby reducing the surface energy. Thus, it can generally be expected that metal‐ALD techniques exhibit island growth behavior following the Volmer–Weber growth mechanism, and films grown through this behavior typically show high roughness characteristics, making the formation of ultra‐thin metal films challenging.

Moreover, nitride materials are also a popular application of ALD technology. Some of these materials (e.g. TiN, TaN, and ZrN) have high electrical conductivity, comparable to metals, while others have very low conductivity, which allows them to be used in a wide variety of applications. For the deposition of nitride materials, NH_3_, N_2_H_4_, NH_3_ plasma, N_2_ plasma, and N_2_/H_2_ mix plasma, which can supply nitrogen, are mainly used as reactants [20, 21, 25, 26, 27, 28, 29, 30, 31].

Sulfide materials can also be deposited by ALD, and there has been increasing interest in related research in recent years. H_2_S and S have been used as reactants for the deposition of sulfide materials. They exhibit semi‐metallic properties, and because they are deposited by ALD, the desired thin films can be obtained at relatively low temperatures. Metal sulfides ALD often follow a ligand exchange mechanism, similar to the metal oxide reaction process.

In the field of catalysis, ALD has received significant attention due to its numerous advantages including possible to deposit a wide variety of materials. The utilization of ALD can lead to performance enhancements in two major aspects. Firstly, ALD coatings are conducted to show the barrier properties for the stabilization and passivation of catalyst particles. The dense ALD film formed on the particle surface provides a unique surface sealing function, which can protect the underlying material from the surrounding environment. Consequently, this enhances the stability of the particles. This technique is mostly conducted using metal‐oxide ALD, with Al_2_O_3_ being the most representative material. Due to its compactness and mature technology, Al_2_O_3_ is widely used as an ALD coating material for surface passivation, protecting against oxidation, high temperature, moisture, and corrosion [33]. Other oxide materials such as SiO_2_, TiO_2_, and ZrO_2_ have also been explored as a surface barrier layer material. Moreover, SiO_2_ demonstrates stability under acidic conditions (pH ≥2) and moderately alkaline conditions (pH ≤ 10). It exhibits excellent electrochemical stability under both reductive and oxidative potentials and maintains high‐temperature resistance, remaining stable up to 650°C. Therefore, SiO_2_ is a suitable material as a protection layer for catalysts. Li et al. [25b]. reported the effect of SiO_2_ ultra‐thin protective layer on catalysts. They demonstrated that the current density of Pt on carbon black catalyst was reduced only by 34% to 7% at −0.2 V versus RHE and Pt detachment/dissolution was reduced by 8.94 to 1.94 mg L^−1^ after an accelerated durability test after just 5 cycles of SiO_2_ ALD cycles. Therefore, a nanoscale SiO_2_ protective layer strongly reduces Pt agglomeration and detachment during the HER in acidic conditions. These results indicate that ALD is a suitable deposition method, offering sub‐nanometer control over layer thickness and the production of uniform, conformal coatings on various substrate surfaces. TiO_2_ is a support material known for its considerable electrochemical activity and improved durability. It exhibits good mechanical resistance and chemical stability in acidic and oxidative conditions. However, TiO_2_ supports face challenges, such as increased resistance at the electrode and higher overpotential compared to untreated catalysts, due to the low conductivity of the thick metal oxide‐based support. Therefore, research is needed to precisely control the thickness of the TiO_2_ protective layer for catalytic activity. Chung et al. [34]. reported that an ultrathin TiO_2_ layer on Pt/C catalyst could maintain the activity of the catalyst and conductivity by blocking the water to carbon physically. From these results, we know that the ALD technology is important in depositing ultrathin and uniform TiO_2_ layers and controlling the exact thickness of the deposited layer on the carbon [34]. ZrO_2_ also was used as the protective layer due to its high chemical and mechanical stability such as excellent corrosion resistance [35, 36]. Chen et al. [37]. reported that an ultrathin ZrO_2_ layer passivated Al NPs to enhance the stability of the NPs. The experimental results of the Al@Al_2_O_3_@ZrO_2_‐8 core–shell structures showed excellent stability in water at 80°C, with 82.2 wt% of metallic Al, maintaining about 93.4% of pure Al NPs. From these results, it can be seen that ALD technology can deposit many kinds of thin films, and ALD thin film coatings of less than 1 nm can form a passivation layer that can have complete oxidation resistance. Additionally, the uniform and conformal surface coverage, along with the precise thickness control provided by ALD, can help minimize the amount of surface passivation material used. This is crucial for applications where it is important to preserve the bulk properties of the original materials as much as possible [33].

On the other hand, ALD is utilized to fabricate highly dispersed, size‐controllable metal NPs to increase the reactive sites for catalytic reactions by forming an island structure of metal particles [38]. This technique is most often performed using metal‐ALD utilizing materials such as Pt, Ru, Ir etc. As mentioned earlier, island growth has difficulty forming ultra‐thin films, however, for the catalysts, the Volmer–Weber growth mechanism could help to increase the performance of catalysts due to the maximized surface area of the metal NP‐based catalysts. In addition, ALD enables uniform deposition of NPs even on porous substrates, resulting in strongly enhanced chemical reactivity due to the high surface area [39]. Based on these results, the metal‐ALD technique can be used to uniformly distribute metal NPs on substrates such as carbon supports and to control their size to maximize catalytic performance while reducing cost. Therefore, synthesizing catalytic materials using oxide and metal ALD will enable the design and controllable fabrication of catalysts with unique structures, resulting in catalysts with improved performance in both stability and activity.

Additionally, many metal nitride materials (such as TiN, MoN, NiMoN, TaN etc.) have distinct advantages towards electrocatalysis owing to their good electrical conductivity, corrosion resistance, and chemical stability [40]. Therefore, if nitride materials are used as hydrogen evolution reaction (HER) catalysts, we can expect catalysts with excellent performance and stability. Ramesh et al. [40]. reported that MoN_x_/3D Ni‐foam composite catalyst exhibited improved HER current density, very high hydrogen evolution capability, and low overpotential value. It was also reported that the stability was improved in both acidic and alkaline electrolytes. Based on these results, it can be concluded that various materials including nitrides and sulfides by utilizing ALD technology can contribute to the improvement of catalyst performance.

### 2.2 Thin and Conformal Coating

To obtain uniformly covered surfaces by applying ultra‐thin film coatings to catalysts, ALD technique with precise thickness control at the nanometer scale is required. In ALD, the adsorption of precursors allows for the formation of thin films with precise thickness and composition, achieved by the layer‐by‐layer deposition of atoms or molecules on the substrate surface [23]. So, it is possible to grow uniform and conformal films on various substrate surfaces [25b].

**FIGURE 4:**
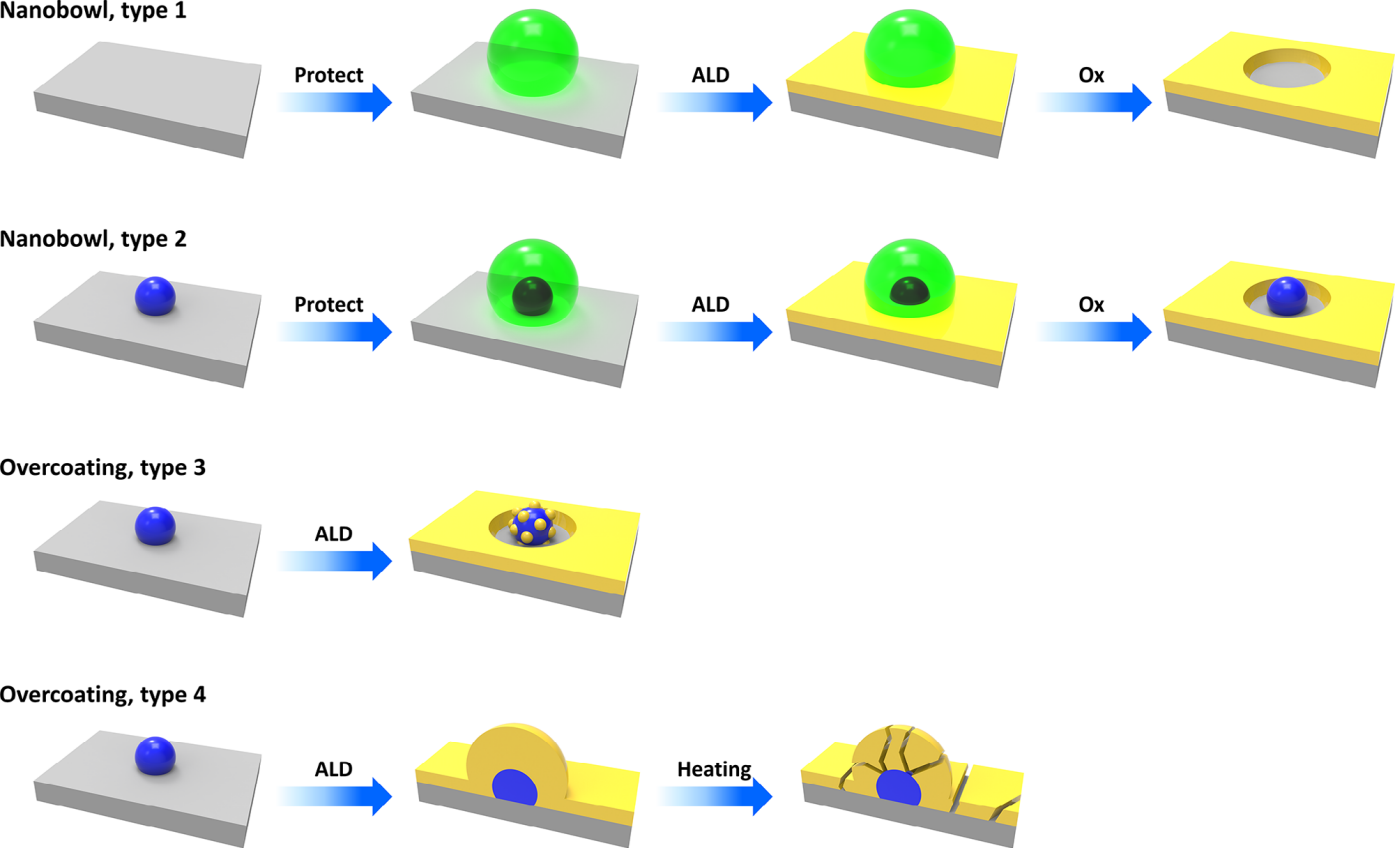
Schematic represents the strategies for altering supported metal NPs using ALD overcoating: nanobowl synthesis; metal NPs isolated in nanobowl; selective decoration of NPs; and complete overlayer coating, followed by heat treatment to induce nanoscale porosity. Gray color signifies the support material, blue represents metal NPs, yellow represents the ALD overcoat, and green exhibits a removable template molecule [21].

In the field of catalysis, catalysts are often destroyed or reconstructed during reactions, which affects their stability. Specifically, catalysts are not always used in the chemically stable electrolyte of water but are also exposed to various types of electrolytes, such as acids and seawater. Therefore, in addition to catalytic efficiency, it is crucial to prevent catalyst corrosion and dissolution by passivating the surface of active catalysts [23]. Ideally, a protective film of adequate thickness would be formed to effectively protect the metal atoms and prevent catalyst poisoning. The ALD technique is particularly well‐suited for applying such a protective film to catalysts. The protective film should have an appropriate thickness, and the optimization of the protective film thickness should be carried out according to the catalyst material to show the best performance. Using ALD technology, the thickness of the coating layer can be precisely controlled by adjusting the number of ALD cycles, allowing for a conformal coating. After applying such an ALD coating, it is expected that the durability and stability of the catalyst will be enhanced while maintaining its activity. Consequently, thin and conformal coatings are increasingly used as corrosion protection layers to improve surface electrical passivation and enhance electrochemical stability. Christos et al. [25] reported that the deposition of thin film coatings and catalyst NPs using ALD on Si photoelectrodes could improve performance and stability. Firstly, they did thin film coatings for electrical passivation of surface defects to improve the performance of the Si photocathodes. Then, Pt NPs were deposited on Si photocathodes to enhance the catalytic performance. Or, after the formation of a film with catalytic properties, annealing at high temperature finally forms a cracked film to increase the ability. Alternatively, additional catalytically active particles such as Pt can be formed after the formation of an ultra‐thin film of a material that acts as a substrate (nanopowder). Moreover, O'Neill et al. [21] demonstrated four different approaches (types 1–4) to engineer the surface of supported metal NP catalysts by taking advantage of ALD as shown in Figure 4. The ALD technique can be used to get the desired shape and size of the supported catalysts Figure 4 (types 1 and 2). In addition, it can be used to prevent agglomeration and dissolution of the supported metal NPs without compromising their catalytic activity (types 3 and 4) (Figure 4). In summary, the stability, activity, and selectivity of supported metal NP catalysts can be improved by offering the ALD overcoat [21].

### 2.3 Coating on Complex Nanostructures

The self‐limiting nature of ALD technology allows for the deposition of films with excellent conformality, even for extremely complex 3D structures and morphologies. Moreover, ALD is insensitive to differences in precursor flux, the growth rate is the same everywhere in the ALD reaction chamber. In contrast, other vapor deposition techniques such as physical vapor deposition (PVD) and chemical vapor deposition (CVD) are flux‐controlled deposition methods, which means that the materials can be deposited mainly along the direction of the flux. In this way, ALD enables conformal film deposition, enabling uniform coating of the desired material, not only on high‐aspect‐ratio trench structures but also on 3D complex nanostructures. In particular, the self‐limiting nature of ALD allows precursor molecules to diffuse into narrow pores until they encounter an active site, resulting in a very uniform coating of the pores, which is very useful for specific catalysis reactions [41].

Furthermore, ALD can be used to fabricate catalysts with uniform control of highly deposited dispersed metal or oxide NPs in the channels of porous materials. This has the added advantage that, unlike traditional liquid‐phase methods, no additional steps are required to remove excess metals, undesired solvents, and other reagents [42]. In Table 2, general features of the various vapor deposition techniques are briefly summarized.

**TABLE 2:** Comparison with other vapor phase deposition techniques [26, 27].

Features	PVD	CVD	ALD
Deposition temperature	Relatively low	High	Moderate to low
Degree of vacuum	High vacuum	Medium	Low
Deposition pattern	Nucleation/Grain growth	Nucleation/Grain growth	Layer‐by‐layer deposition
Uniformity	Fair	Fair	Excellent
Conformality	Fair	Fair	Excellent
Step coverage	Poor	Fair	Excellent
Film thickness	Nanometer‐scale	Nanometer‐scale	Angstrom level
Growth rate	Fast	Medium	Slow
Impurity	Very low (<1%)	Varies	Low (<1%)
Expansibility	Medium	Low	High
Thickness control	Analog (deposition time)	Analog (deposition time)	Digital (number of ALD cycle)
Composition control	Type of target/Reaction gas ‐ Relatively narrow uniform composition in multi‐component films	Partial pressure ratio of reactive gas ‐ Local composition non‐uniformity ‐ Hard to control composition in multi‐component films	Digital ‐ Super‐cycle structure ‐ Precise composition control ‐ Possible to design a non‐existing material
Application	Pure films and durable coatings ‐ Electronics ‐ Optics ‐ Automotive	Precise control of film composition and properties ‐ Semiconductor manufacturing ‐ Thin‐film deposition ‐ Surface engineering	Atomic‐level precision in thin‐film deposition ‐ Semiconductor device fabrication ‐ Advanced coatings ‐ Nanotechnology

### 2.4 Area‐Selective or Site‐Selective ALD Deposition

Area‐selective ALD (AS‐ALD) is known as a promising method to form unique nanostructures by precisely controlling the growth of specific components on a specific type of site. In addition, materials can be selectively deposited on a substrate according to the desired purpose. Thus, AS‐ALD can be utilized to form 3D nanostructures with spatial control, such as covering only certain faces, edges, or corners of the catalyst particles with functional elements [38].

**FIGURE 5:**
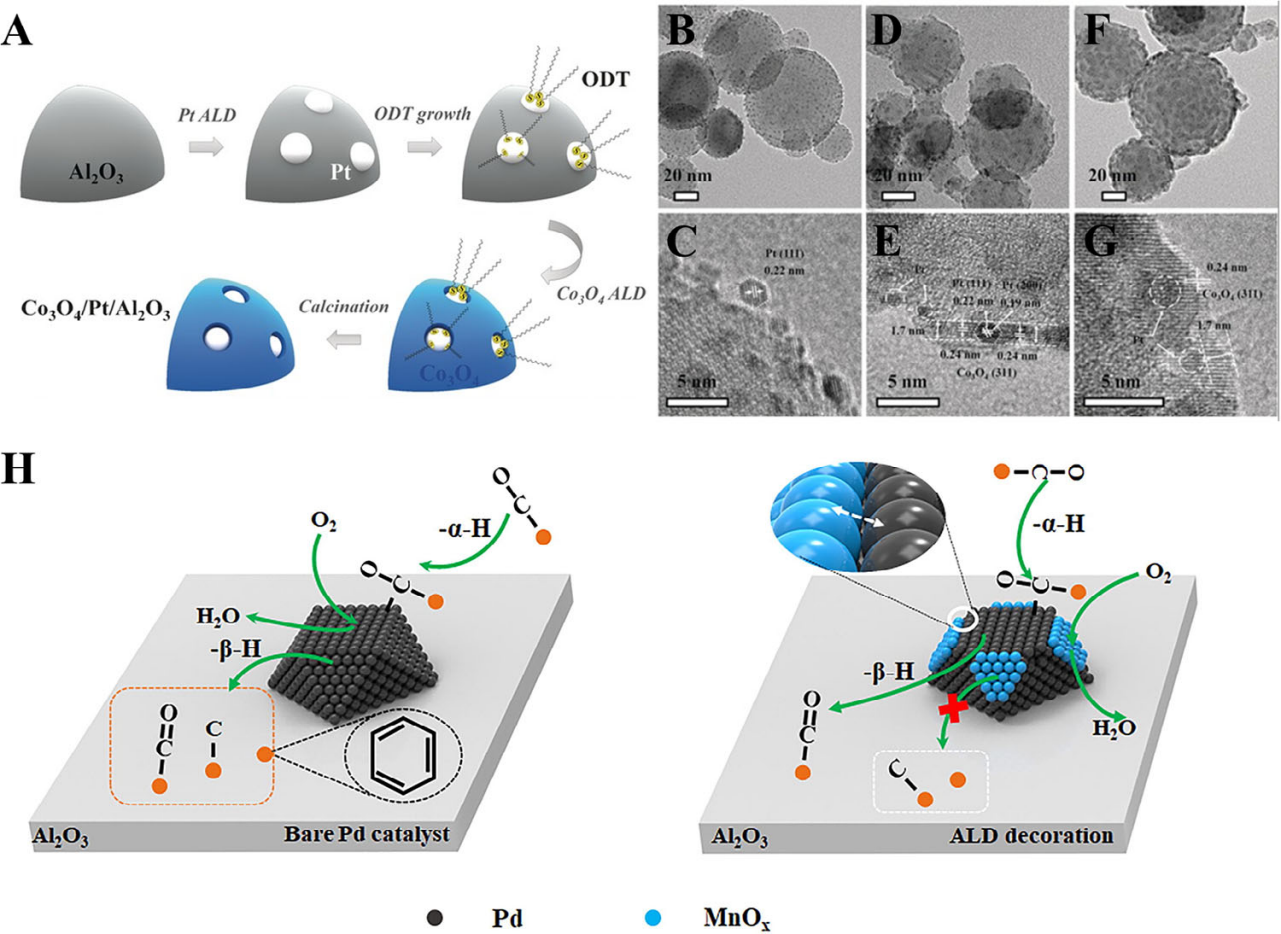
(A) Schematic drawing for synthesizing Co_3_O_4_ nanotrap‐anchored Pt NPs on Al_2_O_3_ support by AS‐ALD. (B,C) TEM images for Pt/Al_2_O_3_; (D,E) Co_3_O_4_/Pt/Al_2_O_3_ (Co_3_O_4_ nanotrap‐anchored Pt NPs); and (F,G) Co_3_O_4_@Pt/Al_2_O_3_ (Co_3_O_4_ overcoated Pt NPs). Reproduced with permission [44a]. Copyright 2016, Wiley‐VCH. (H) Schematic presentation for the site‐selective ALD‐deposition of MnO_x_ on Pd/Al_2_O_3_. Reproduced with permission [44b]. Copyright 2020, Elsevier.

The selective deposition of the desired material in these ALD techniques results from the rapid growth behavior of the material at specific sites or surfaces, while nucleation delays occur initially in passivation regions such as sites or substrates where deposition is not desired. The AS‐ALD is driven by the appropriate combination of precursor, reactant, substrate type, precursor partial pressure, deposition temperature etc. Alternatively, pretreatment can be performed before the ALD technique to selectively functionalize certain parts of the substrate or sample. In other words, if self‐assembled monolayers, electron or ion‐beam, polymer resist, plasma etc. are used initially, the patterned areas on the substrate are activated or passivated by each substance. Then, once the deposition is done, we can either deposit the desired material where we want it to be deposited, or we can prevent it from being deposited. In most AS‐ALD techniques, organic ligands are used as metal surface passivation layers, which ultimately serve to prevent the deposition of oxide materials during the ALD technique [43, 44]. In other words, they prevent oxide materials from being deposited on the surface of metal NPs during the ALD technique and allow them to be selectively deposited on the support of other types of oxide materials. For example, Pt NPs anchored in the Co_3_O_4_ nanotraps on alumina supports is prepared based on As‐ALD as shown in Figure 5A‒G [44a]. Moreover, AS‐ALD can be used to passivate specific facets of metal catalysts by selectively depositing metal oxides. For instance, selective passivation of Pd (111) facet by decorating MnO_x_ was performed by the AS‐ALD technique as demonstrated in Figure 5H for achieving high selectivity towards specific chemical reaction [44b].

In addition, in the case of AS‐ALD, the oxide material can be deposited directly on the metal catalyst. The reactivity of the precursor directly influences the growth behavior of the oxide material on the metal surface. In the case of the Al_2_O_3_‐ALD technique using TMA as a precursor and H_2_O as a reactant, Al_2_O_3_ tends to favor nucleation at the edges and low‐coordination sites of metals such as Pt or Pd in the initial ALD cycle. However, as ALD cycles increase, a continuous Al_2_O_3_ film forms and covers the entire substrate surface. In addition, certain crystal orientations can also make a difference in the growth behavior of certain materials. For example, CeO_x_ species can also be selectively nucleated, as they are favored to nucleate on Pt(111) but not on Pt(100).

**FIGURE 6:**
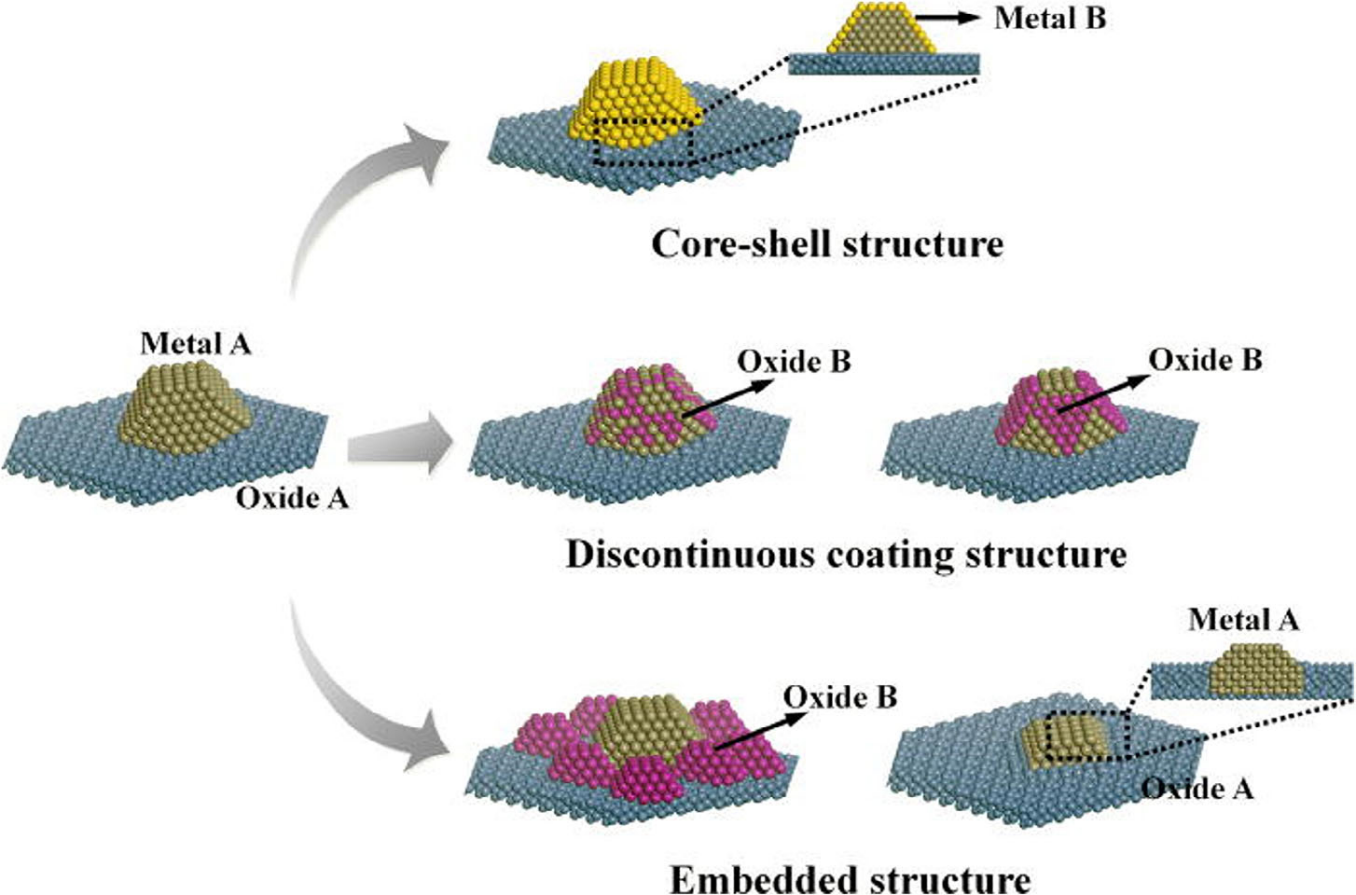
The schematic drawing represents the different composite catalysts such as core–shell, discontinuous coating, and embedded structures by selective ALD technique [38].

In other words, AS‐ALD can be utilized to produce nanostructured composite catalysts with controlled structures, such as core–shell, discontinuous coating, and embedded structures as shown in Figure 6 [38]. Moreover, it can be used to design highly selective and efficient catalysts for specific applications by controlling structural parameters, interfaces, and active sites of the support materials. Both the selectivity and activity of the catalysts have recently attracted great attention in the field of catalysis for energy applications [38, 43, 45].

## 3 Critical Aspects of Catalysts

**FIGURE 7:**
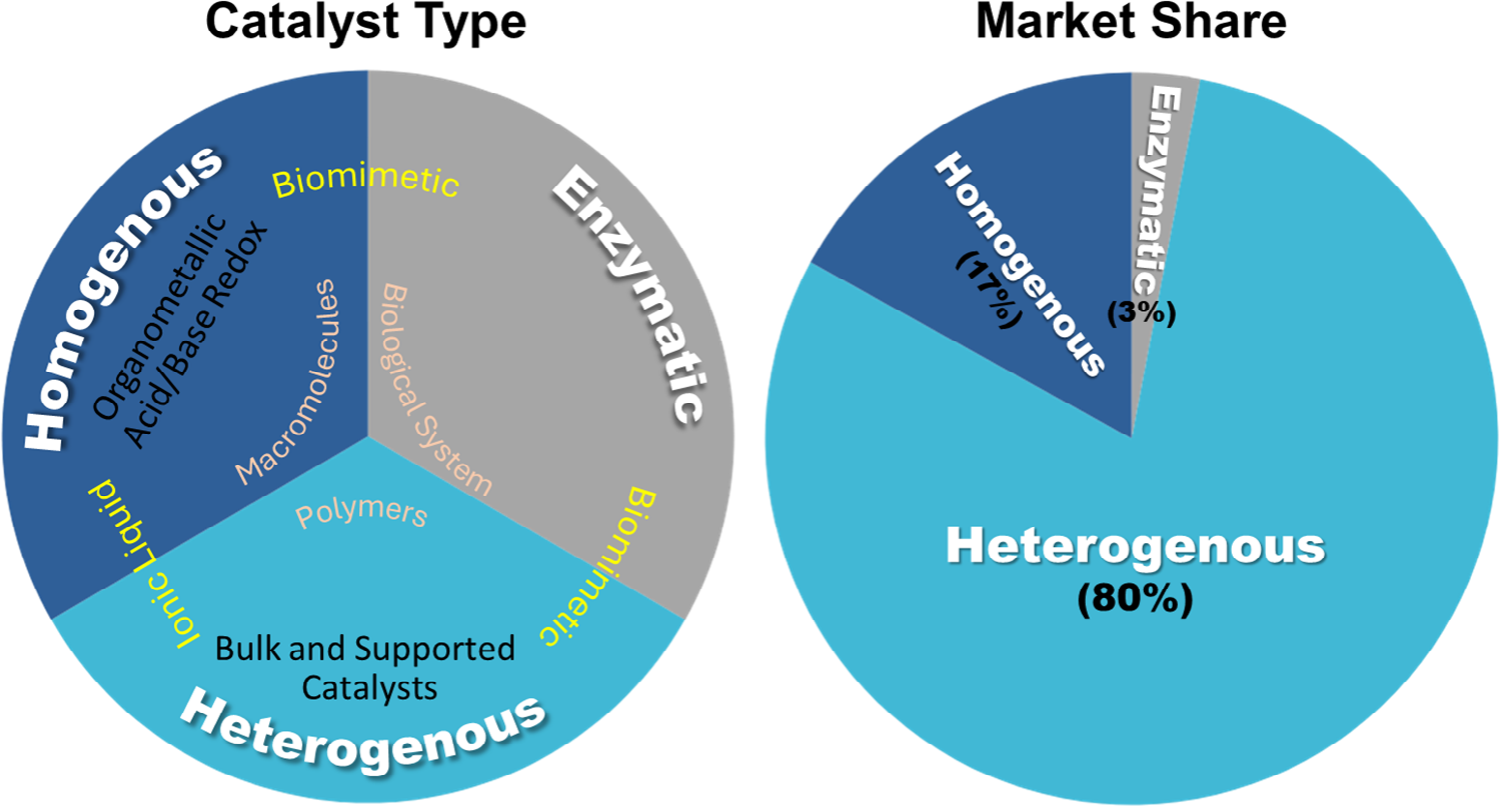
Pie charts represents various types of catalysts and their global industrial usage.

Catalysts are used to enhance the formation rate of targeted products. The desired products would not either form or form extremely slowly in the absence of catalysts. Catalysts are explored in diverse fields such as biological and non‐biological processes. The catalysts are two types biological and non‐biological. The biological catalytic route is termed as enzymatic catalysis and the non‐biological catalysts (e.g. organic or inorganic materials) are generally named as homogeneous or heterogeneous catalysis. In homogeneous catalysis both the catalysts and reactants remain in same phase (e.g. liquid and/or gas phase) whereas different phases are involved in the heterogeneous catalysis. Organometallic compounds are commonly used in homogeneous catalysis for industrial processes like hydrogenation, hydrocyanation, olefin metathesis, alkene polymerization, alkene oligomerization, hydrocarboxylation, methanol carbonylation, and hydroformylation by oxidative addition and reductive elimination. Various supported‐metal catalysts (e.g. carbon supported Pt‐metal catalyst) are used as heterogeneous catalysis for chemical processing and energy conversion and storage applications. In industrial catalytic processes, heterogeneous catalysts are predominantly used (80%) compared to homogeneous (17%) and enzymatic (3%) catalysis (Figure 7) [46]. This review particularly discusses the heterogeneous catalysis for energy generation, conversion, and storage applications. More specifically, we emphasize the recent development of heterogeneous catalysts by the ALD technique for electrochemical and photochemical energy conversion and storage applications unless otherwise mentioned in this article. We carefully elaborate on the critical aspects of energy‐related catalysts (e.g. catalyst design, synthesis and characterization, surface science of catalysts, and reaction kinetics) and discuss their recent development by the ALD technique. Electrochemical energy conversion is highly dependent on the efficiency of catalysts and their reaction kinetics. An electrochemical reaction involves an oxidation and reduction reaction performed on separated half‐cells [47]. Like other chemical reactions, in the case of electrocatalysts, it is also necessary to provide adequate activation energy which is known as overpotential [48]. The role of an electrocatalyst is to minimize the overpotential by tuning the free‐energy landscape between the initial and final states [49]. Therefore, designing and fabricating a suitable electrocatalyst plays a key role in enhancing the efficiency of energy conversion. The performance of the particular electrocatalyst is highly dependent on the type of catalyst, reaction kinetics, and preparation methods.

### 3.1 Types of Catalysts

Up to date, numerous heterogeneous catalysts such as metal/metal alloy‐based, metal oxide‐based, metal sulfide‐based, metal nitride‐based, metal carbide‐based, and carbon‐based catalysts have been explored for electrochemical and photochemical energy conversion and storage applications (Table 3) [50, 51, 52, 53, 54, 55, 56, 57, 58, 59, 60, 61, 62, 63, 64, 65, 66, 67, 68, 69, 70, 71, 72, 73, 74, 75, 76, 77, 78, 79, 80, 81, 82, 83, 84, 85, 86, 87, 88, 89, 90, 91, 92, 93, 94, 95, 96, 97, 98, 99, 100, 101, 102, 103, 104, 105, 106, 107, 108, 109, 110, 111, 112, 113, 114, 115, 116, 117, 118, 119, 120, 121, 122, 123, 124, 125, 126, 127, 128, 129, 130, 131, 132, 133, 134, 135, 136, 137, 138].

**TABLE 3:** Different types of heterogeneous catalysts for energy conversion, generation, and storage applications.

Types of catalyst	Functional materials	Catalysis	Applications	Ref.
Metal and metal alloys‐based catalysts	Six Pt‐group metals on the carbon support	Electrocatalysis Fuel cells	H_2_ fuel generation Electricity generation	[50]
	PtRu alloy on C_2_N matrix	Electrocatalysis	H_2_ fuel generation	[51]
	IrW alloy on carbon	Electrocatalysis	H_2_ fuel generation	[52]
	NiRu alloy coated with graphitic carbon	Electrocatalysis	H_2_ fuel generation	[53]
	Pt_3_Ni on carbon support	Electrocatalysis Fuel cells	H_2_ fuel generation Electricity generation	[54]
	NiMo alloy on Ni‐foam	Electrocatalysis	H_2_ fuel generation	[55]
	NiTi alloy on Ti‐felt	Electrocatalysis	H_2_ fuel generation	[56]
	Pt on carbon support	Fuel cells	Electricity generation	[29]
	Ternary PtCoCu alloy	Fuel cells	Electricity generation	[57]
	PtNiCo alloy on N‐doped carbon support	Fuel cells	Electricity generation	[58]
	PtMo on carbon support	Fuel cells	Electricity generation	[59]
	PtFe nanoalloys supported on Fe‐based cubic framework	Fuel cells	Electricity generation	[60]
	Defective CuNi alloy	Electrocatalysis	Water splitting	[61]
Metal oxide‐based catalysts	IrO_2_ decorated W_18_O_49_ nanowire	Electrocatalysis	Water splitting	[62]
	Mn‐doped NiO supported on rGO	Photo electrocatalyst	Photo electrocatalytic hydrogen generation	[63]
	CuO@MnO_2_ on N‐doped CNT	Electrocatalysis	Supercapacitor	[64]
	Layered g‐C_3_N_4_/TiO_2_	Photo electrocatalyst	Photo electrocatalytic hydrogen generation	[65]
	CoO‐hydrogenated TiO_2_ nanosheets	Photo electrocatalyst	Photo electrocatalytic hydrogen generation	[66]
	Oxygen defects rich Co_3_O_4_ nanoribbons	Electrocatalysis	Supercapacitor	[67]
	CuCo_2_O_4_ porous nanosheets	Electrocatalysis	Supercapacitor	[68]
	Hybrid nanocomposite of CNT‐Mn_3_O_4_/CoWO_4_	Electrocatalysis	Battery	[69]
	Composite of (Co, Mn)_3_O_4_/N‐doped CNT	Electrocatalysis	Battery	[70]
	Hollow microspheres Co_3_V_2_O_8_	Electrocatalysis	Battery	[71]
	TiO_2_─CeO_2_ supported on rGO	Photo electrocatalyst	Photo electrocatalytic hydrogen generation	[72]
	B‐doped IrO_2_─Ta_2_O_5_	Electrocatalysis	Water‐splitting	[73]
	Nanoribbon monoclinic IrO_2_	Electrocatalysis	Water‐splitting	[74]
	IrO_2_ supported on hollow Co_3_O_4_─CoMoO_4_	Electrocatalysis	Water‐splitting	[75]
	IrO_2_ cluster on MnO_2_	Electrocatalysis	Water‐splitting	[76]
	Monoclinic Ir_x_Ru_1−x_O_2_	Electrocatalysis	Water‐splitting	[77]
	Mo‐doped mesoporous RuO_2_	Electrocatalysis	Water‐splitting	[78]
	RuO_2_─CeO_2_ on carbon cloth	Electrocatalysis	Water‐splitting	[79]
	RuO_2_ on Ni foam	Electrocatalysis	Water‐splitting	[80]
	NiO/NiCo_2_O_4_/Ni foam	Electrocatalysis	Water‐splitting	[81]
	NiCo_2_O_4_@CeO_2_	Electrocatalysis	Water‐splitting	[82]
Metal sulfide‐based catalysts	Spinel NiCo_2_S_4_	Electrocatalysis	Water‐splitting	[83]
	MoS_2_ on leaf‐like CdS	Photo electrocatalyst	Photo electrocatalytic hydrogen generation	[84]
	Composite of CoS_2_─FeS_2_─N doped carbon	Electrocatalysis	Battery	[85]
	Composite of CuCoS@NiCoS─N doped carbon	Electrocatalysis	Battery	[86]
	Composite of NiS_2_‐MnS on MoS_2_/N‐doped graphene	Electrocatalysis	Battery	[87]
	Co_3_S_4_ with amorphized surface	Electrocatalysis	Supercapacitor	[88]
	ZIF derived MoS_2_/Co_3_S_4_/Carbon	Electrocatalysis	Supercapacitor	[89]
	MoS_2_ nanoflower/g‐C_3_N_4_	Photo electrocatalyst	Photo electrocatalytic hydrogen generation	[90]
	NiCuInS_2_:In_2_S_3_/Graphitic nitride composite	Photo electrocatalyst	Photo electrocatalytic hydrogen generation	[91]
	Petal‐like Fe_x_S_y_/WS_2_ nanosheets	Photo electrocatalyst	Photo electrocatalytic hydrogen generation	[92]
	La‐doped Ni_3_S_2_/MoS_2_ on Ni‐foam support	Electrocatalysis	Water‐splitting	[93]
	Cu_2_S─MoS_2_ on carbon cloth	Electrocatalysis	H_2_ fuel generation	[94]
Metal nitrides‐based catalysts	Ternary metal (Ni, Fe, Cr) nitride on N‐doped carbon support	Electrocatalysis	Water‐splitting	[95]
	Co_5.47_NMoN on Ni foam support	Electrocatalysis	Water‐splitting	[96]
	Pt─Ni@NiMoN on Ni foam support	Electrocatalysis	H_2_ fuel generation	[97]
	Ru‐exsolved TiN nanotubes	Electrocatalysis	H_2_ fuel generation	[98]
	W_2_N_3_/Fe_2_N	Electrocatalysis	Water‐spilling	[99]
	WN/g‐C_3_N_4_	Photo electrocatalyst	Photo electrocatalytic hydrogen generation	[100]
	Defective MoN/g‐C_3_N_4_	Photo electrocatalyst	Photo electrocatalytic hydrogen generation	[101]
	Mo_2_N/Ni_0.2_Mo_0.8_N nanobelts	Electrocatalysis	H_2_ fuel generation	[102]
	Oxygen‐modulated MoN_x _cluster on carbon sphere	Electrocatalyst	Battery	[103]
	Multi‐branched vanadium nitride	Electrocatalyst	Battery	[104]
	Ni─Mo─N on stainless steel mesh	Electrocatalyst	Supercapacitor	[105]
	Ni_3_N‐CoN/N‐doped carbon/carbon cloth	Electrocatalyst	Supercapacitor	[106]
	Pt loaded on boron nitride sheets	Electrocatalysis Fuel cells	Electricity generation	[107]
	Pt supported on porous CrN nanogrids	Electrocatalysis Fuel cells	Electricity generation	[108]
	Ru/BN@C	Electrocatalysis	H_2_ fuel generation	[109]
	Co_3–x_Fe_x_Mo_3_N	Electrocatalysis	Water‐splitting	[110]
Carbon‐based catalysts	Ru nanocluster on defective g‐C_3_N_4_	Electrocatalysis	Water‐splitting	[111]
	Pt single atom on g‐C_3_N_4_	Photo electrocatalyst	Photo electrocatalytic hydrogen generation	[112]
	Cu single atom on g‐C_3_N_4_ nanotube	Photo electrocatalyst	Photo electrocatalytic water splitting	[113]
	Dual Ni‐Mn atoms on N‐doped porous carbon support	Electrocatalysis	Energy conversion (CO_2_ conversion)	[114]
	Nb single atom anchored on N‐doped carbon	Electrocatalysis	Battery	[115]
	Fe─Co dual atom on N‐doped carbon	Electrocatalysis	Battery	[116]
	Fe─N─C single derived from ZIF	Electrocatalysis	Battery	[117]
	Fluorine, nitrogen doped carbon	Electrocatalysis	Water‐splitting	[118]
	Carbon dots anchored on porous N‐doped carbon	Electrocatalysis	Supercapacitor	[119]
	Oxygen‐doped graphitic nitride	Electrocatalysis	Supercapacitor	[120]
	Carbon dots embedded on g‐C_3_N_4_	Photo electrocatalyst	Photo electrocatalytic hydrogen generation	[121]
	Ni single atom on N‐ doped graphene nanofiber	Electrocatalysis	Energy conversion (CO_2_ conversion)	[122]
	Pt single atom on 3D porous carbon	Fuel cells	Electricity generation	[123]
New class of catalysts	Pt‐Co single atom alloy supported on N‐doped graphitized carbon nanotubes	Electrocatalysis Fuel cells	Electricity generation	[124]
	NiCo_2_O_4_ on MXene	Electrocatalysis	Water‐splitting	[125]
	Crystalline g‐C_3_N_4_/2D TiO_2_ on Ti_3_C_2_T_x_	Photo electrocatalyst	Photo electrocatalytic hydrogen generation	[126]
	PtCo NPs and single atoms on N‐doped graphene nanofiber	Electrocatalysis Fuel cells	Electricity generation	[127]
	Ru/In dual and single atom supported on TiO_2_	Photo electrocatalyst	Photo electrocatalytic hydrogen generation	[128]
	Co_S_/FeS_2_ on MXene	Electrocatalysis	Water‐splitting	[129]
	Pt single atom on boron nitride	Fuel cells	Electricity generation	[130]
	FeNi‐layered double hydroxides arrays supported on Ti_3_C_2_T_x_	Electrocatalysis	Supercapacitor	[131]
	N‐Ti_3_C_2_T_x_/sulfur composite on rGO	Electrocatalysis	Battery	[132]
	High entropy‐MXene (TiVNbMoC_3_) doped graphene composites	Electrocatalysis	Battery	[133]
	CoNi dual atom anchored on Ti_3_C_2_T	Fuel cells	Water‐splitting	[134]
	La(CoCrFeMnNiAl_x_)_1/(5+x)_O_3−δ_	Electrocatalysis	Supercapacitor	[135]
	Pt rich shell on N‐doped PtCoFeNiCu high entropy alloy	Electrocatalysis Fuel cells	Electricity generation	[136]
	(Ru_0.2_Ir_0.2_Cr_0.2_W_0.2_Cu_0.2_)O_2_	Electrocatalysis	Water‐splitting	[137]
	(ZnCoMnFeAlMg)_9_S_8_	Electrocatalysis	Water‐splitting	[138]

**FIGURE 8:**
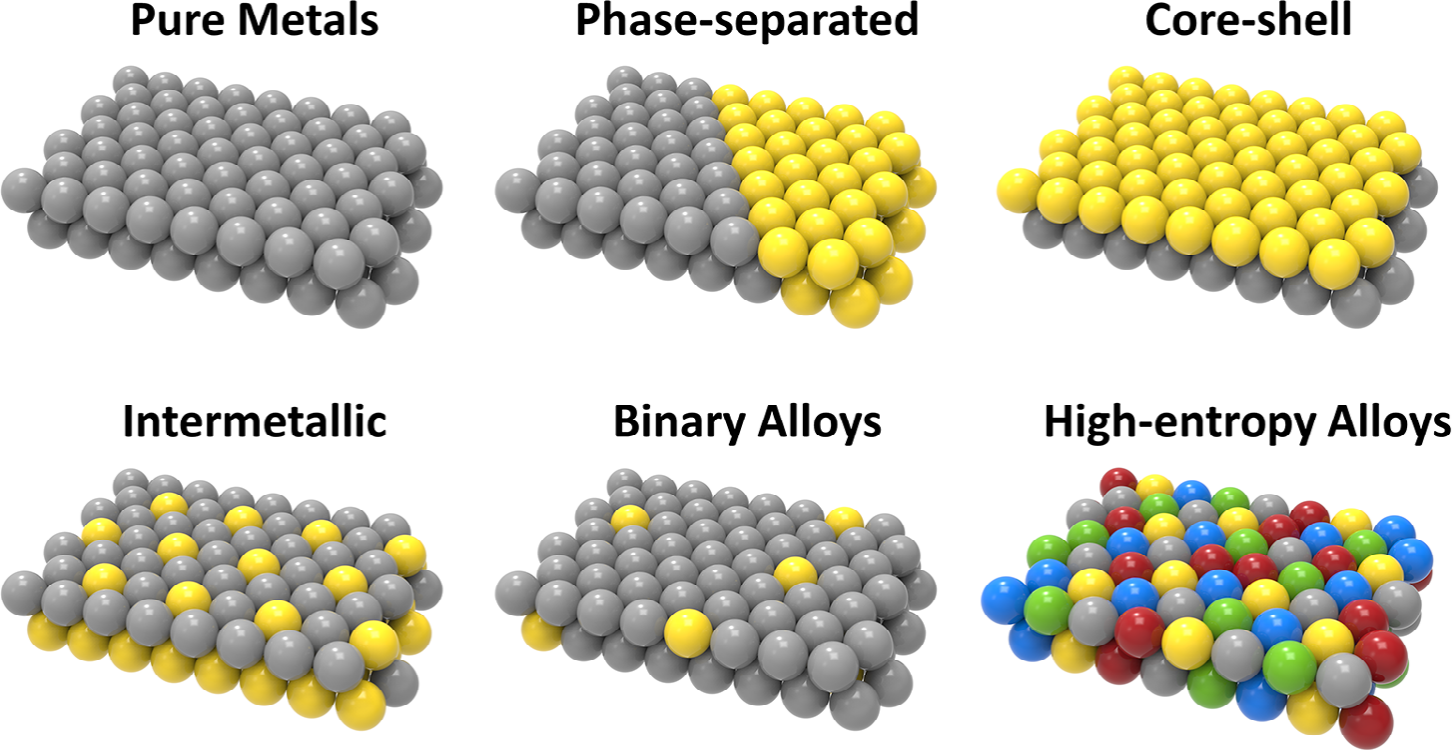
The schematic drawing represents the structure of different binary and multinary alloys including pure metals.

Among the diverse catalysts mentioned in Table 3 for electrochemical energy conversion and storage, Pt group metal (PGM)‐based catalysts are far ahead of other known electrocatalysts in terms of both activity and durability [139]. The surface of the PGM‐based electrocatalysts has moderate free energy for adsorbing and desorbing reaction intermediates which leads to their superior electrocatalytic performance [140]. Despite the outstanding electrochemical properties of the PGM‐based catalysts, the ultra‐high cost is a serious limitation for their large‐scale use. To tackle that issue, some Pt‐based alloys (binary or multinary alloys) with other noble metals and non‐noble metals have recently been developed [140, 141, 142]. Compared to binary alloys multinary metal alloys where different constituent metals provide different active sites and balanced binding energies for reaction intermediates, resulting in improved electrocatalytic properties [142]. Figure 8 represents the structure of different binary and multinary alloys including pure metals. Typically, alloying with other metals results in activity and stability due to synergistic effects and modulation of the electronic structure. Therefore, developing earth‐abundant metal‐based catalysts has recently been a hot topic for researchers to tackle the price and scarcity of noble metal‐based catalysts.

### 3.2 Recent Advancement of Energy‐Related Catalysts

To design an efficient electrocatalyst, composition, geometry, and catalyst utilization aspects should be considered, in the first place, the instinct activity of the selected catalyst has high impact on the overall performance [143]. Since the larger exposed area results in a higher reaction rate, the electrochemical surface area is a key parameter [144]. surface area could be maximized by even distribution and fine size of the catalyst and also porous structure. On the other hand, small particles always have a high tendency to agglomerate, aggregation ends in coarsening and lowering the efficiency [145]. To make sure of uniform distribution of the catalyst and stabilizing fine particles, conductive support with a large surface area and good strong bonding with the catalyst is necessary. Moreover, a porous structure helps improve catalytic activity [146].

Regarding support materials, recently Ti_3_C_2_T_X_ (most common type of MXene) and carbon‐based materials like graphene and carbon nanotube (CNT) have become popular due to high surface area, high conductivity and chemical stability [147, 148, 149]. PtFe‐ordered alloy supported on Ti_3_C_2_T_X_ exhibited superior oxygen reduction reaction (ORR) in comparison with Pt/C because of ordered structure of PtFe and excellent dispersion of PtFe particles on Ti_3_C_2_T_X_ [150].

Moreover, NiCo, NiFe, and CoFe captured in the carbon network also showed great bifunctionality of ORR and Oxygen evolution reaction (OER) because of porous structure and chemical stability of graphene layer on metallic alloys [151]. Unzipping and functionalizing CNTs resulted in forming graphene nanoribbons (GNRs) with high loading of Ni single atoms [149]. In addition, high entropy materials have recently been turned into an exciting topic in the electrocatalyst field. High entropy alloys are defined as multi‐component compounds with more than five elements in which the atomic of each element is between 5 and 35. High entropy materials bring the benefit of thermodynamically stabilizing the solid solution phase by entropy effect. Additionally, the existence of more than five elements in the solid solution phase could create different active sites with short reaction paths to accelerate the overall reaction. Inspired by high entropy alloys, other high entropy materials such as high entropy oxides have been studied as electrocatalysts [152]. Efficient electrocatalysts of PtRhNiFeCu/C [152] toward HER and ORR and (Ru_0.2_Ir_0.2_Cr_0.2_W_0.2_Cu_0.2_)O_2_ [137] toward OER exhibited noticeable activity due to factors such as moderating electron structure, increasing the number of active sites, and the cocktail effect of high entropy materials. Development of the diverse catalysts mentioned above is required to boost the electrocatalytic performance. However, stability, activity, and selectivity of the catalysts are greatly dependent on their geometry and homogeneous dispersion that can be precisely controlled by selecting a suitable preparation method. Therefore, various synthesis methods for developing catalysts are discussed in the next section.

### 3.3 Synthesis Methods for Energy‐Related Catalysts

**FIGURE 9:**
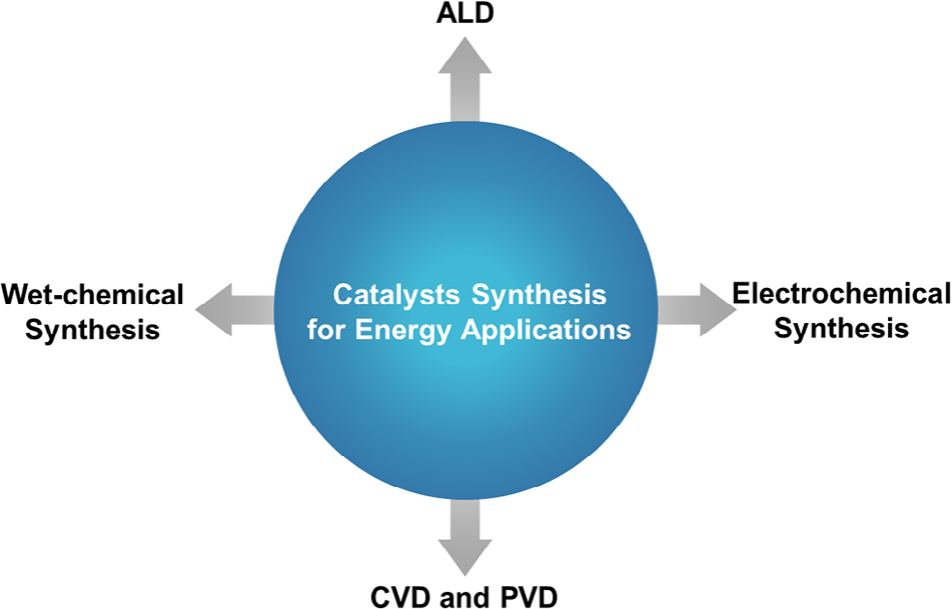
Schematic represents the synthesis methods of catalysts for energy application.

Wet‐chemical synthesis including hydrothermal and solvothermal methods are the most classical and simple ways for synthesizing various catalysts in solution phases [153, 154, 155, 156, 157, 158, 159, 160]. In the wet‐chemical methods, all the chemical reactions of precursor materials and reactant are carried out in particular solvents under appropriate experimental conditions. Moreover, vapor deposition techniques (generally refer to a certain chemical or physical reaction of a material under gaseous phase) such as CVD and PVD are also widely used for developing various nanostructured electrocatalysts for generating clean energy. In vapor deposition technique, the precursor materials in a vapor state are generally going through condensation and chemical reaction to form solid catalysts. The vapor techniques are largely used for functional surface coatings with excellent microstructure [161, 162, 163, 164, 165]. It is important to note that efficiency of electrocatalysts is intimately dependent on their geometrical parameters. Moreover, uniform dispersion and homogeneity are also highly important in achieving high efficiency of the catalysts. However, all these methods limit the preciseness in catalysts deposition, homogeneous dispersion, and geometry of the catalysts. In addition, none of the above‐mentioned methods can atomically control the distribution of the deposited catalysts and/or thickness of thin‐film catalysts. In this regard, ALD is a powerful technique to deposit catalyst precisely on supports and thin films on an atomic scale even on complex surfaces. Such abilities of interface engineering of catalysts at the atomic scale and depositing pinhole‐free thin films are beneficial in the field of energy conversion and storage applications [166, 167]. The major synthetic methods for energy‐related catalysts are schematically shown in Figure 9. Various types of catalyst mentioned in Sections 3.1 and 3.2 have been researched by ALD technique for electrochemical energy conversion and storage applications which are discussed in the next section.

## 4 ALD in Energy Conversion and Storage Applications

**FIGURE 10:**
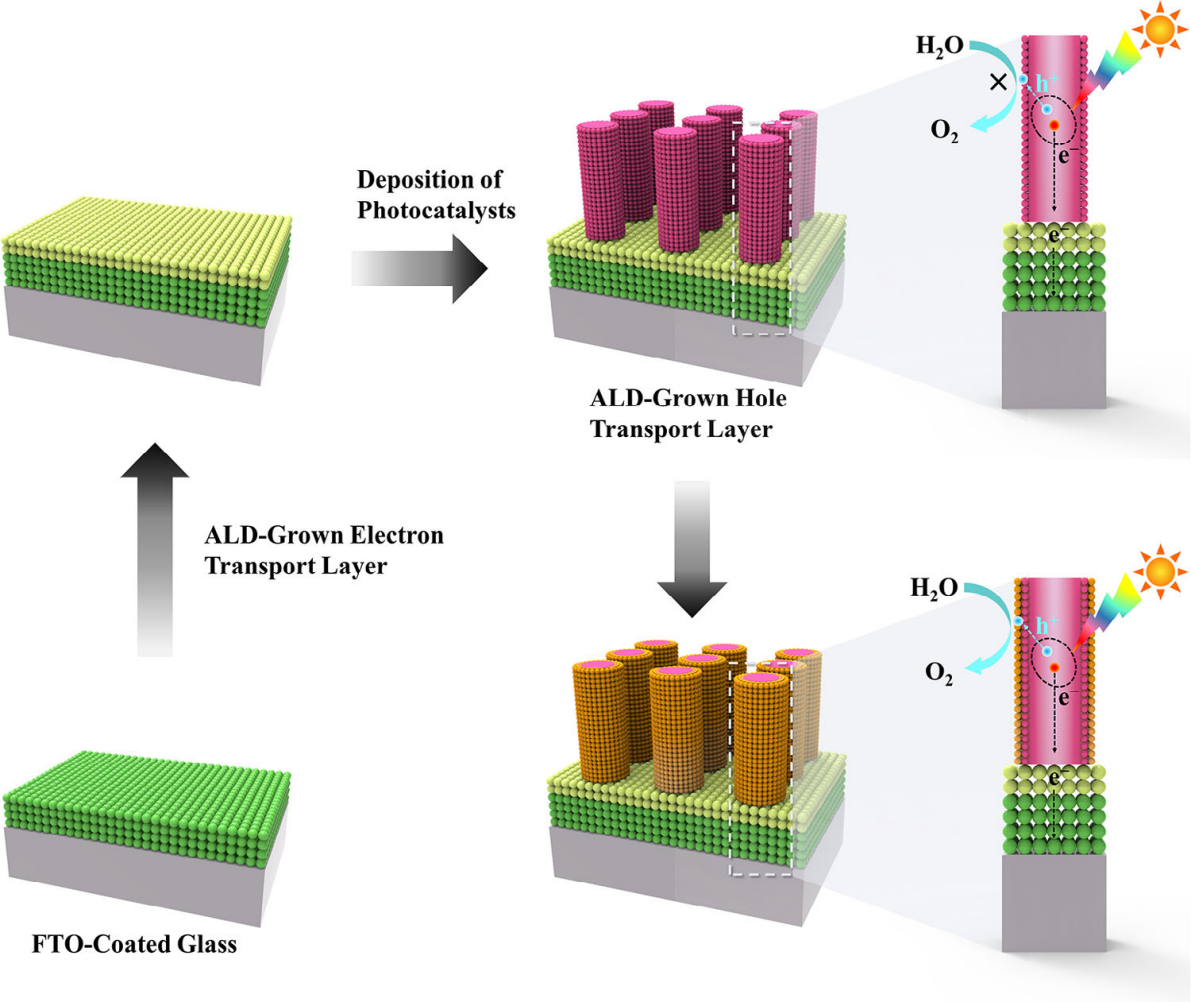
Schematic represents the surface and interface engineering of a photoelectrode for improving its photogenerated electrons and holes transfer properties through the ALD‐grown electron transport and hole transport layers, respectively.

ALD is a well‐known and established technique for thin film fabrications with controlled thickness and interface properties [20]. Compared to other vapor deposition techniques such as CVD and sputtering, the ALD is advantageous and capable of precise catalysts deposition [20]. In the field of energy conversion and storage applications such as photoelectrocatalysis, electrocatalysis, solar cells, fuel cells, batteries, and supercapacitors, ALD is extensively used to atomistically engineer the catalysts to boost their efficiency [21]. For improving solar‐to‐fuel conversion efficiency, surface and interface engineering strategies of a photoelectrode by ALD technique have been schematically shown in Figure 10.

Besides the deposition of conformal coatings and compact thin films, ALD is also explored extensively for the deposition of NPs in modern technology. Supported metal catalysts have attracted considerable attention in the field of electrocatalysis for energy generation in the last decade [14, 15, 20]. It is possible to deposit supported NPs by allowing the nucleation‐controlled growth of metal‐ALD techniques. However, the metal‐ALD techniques require an appropriate choice of metal precursor, substrate, reactant, and ALD conditions [14, 15, 21]. In the case of supported NP deposition by ALD, initially, homogeneously dispersed metal clusters or islands are formed on the surface of the support materials owing to the insufficient chemisorption sites available on support for the precursor molecules and by the virtue of metals as a crystalline nature [15]. The growth of these metal clusters or nuclei on the support proceeds through the surface diffusion phenomena and the predominant interaction of metal precursor with the pre‐deposited metal over the support [14, 15, 16, 17, 18, 19, 20]. The main advantage of metal‐ALD is the nucleation‐controlled growth of metal atoms, which enables atom precise deposition of metal catalysts and makes this technique attractive for the preparation of supported metal NP for catalytic applications [13, 14]. Moreover, the sequential or layer‐by‐layer deposition features of ALD allow for control of the size and loading of catalysts by tuning the total number of ALD cycles [20, 21]. Furthermore, it is possible to deposit target materials and spatially control their deposition over the nanostructures at the atomic scale by taking advantage of area‐selective ALD. For example, core–shell nanomaterials, embedded NPs, and nanoalloys can be synthesized by this selective deposition feature of ALD [2, 20, 21]. It is noteworthy to mention that such core–shell nanostructured materials, embedded NPs, and nanoalloys have been explored extensively as heterogeneous catalysts, particularly in the field of energy conversion and storage applications, and have exhibited outstanding performance [2, 3, 20, 21]. In the following subsections, we have demonstrated the recent advancement of ALD in synthesizing and engineering the electronic structures of the developed catalysts for specific energy conversion and storage applications. Moreover, we have discussed the necessity of precise control over nanostructures, surface, and interface of the catalysts for atomistic modulation of a particular reaction, and how ALD can defeat other vapor deposition or wet‐chemical techniques as far as the precision in materials synthesis is concerned.

**FIGURE 11:**
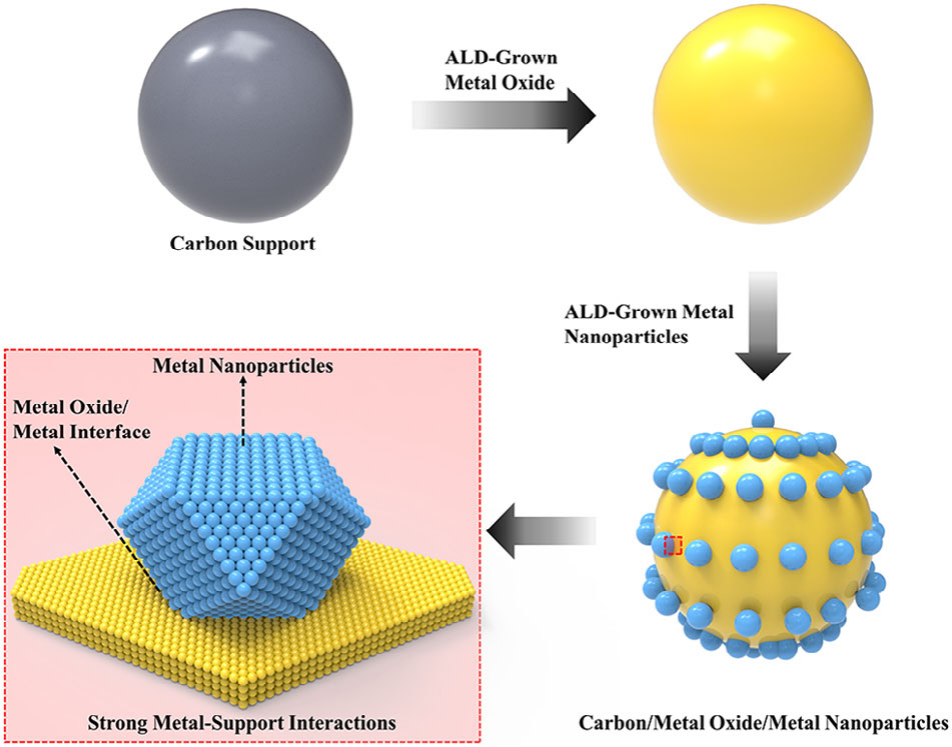
Schematic illustrates the interface engineering of a conventional electrocatalyst comprising metal nanoparticles as catalyst, carbon support, and metals oxide as interface layer between carbon support and catalyst. The rectangle‐shaped red marked area in the schematic for “carbon/metal oxide/metal nanoparticles” represents interface between metal oxide and metal NPs. The strong metal‐support interaction arises due to the chemical interaction between metal oxide layer and metal NPs, leading prevention of migration or agglomeration of metal NPs.

ALD technique has been extensively used in designing efficient catalysts for energy conversion and storage applications such as electrocatalysis, fuel cells, batteries, and supercapacitors including solar energy conversion technologies such as solar cells and PEC cells [2]. ALD has shown unique merits in designing efficient and durable electrocatalysts by tailoring the surface/interface of catalysts and inducing strong catalyst‐support interactions (Figure 11) that are required to prevent detachment, migration, and aggregation of catalyst nanocrystals during electrochemical reactions [2, 20]. Moreover, ALD has been actively used in battery and supercapacitors technologies due to its unique advantages such as self‐limiting growth, precise control over thickness, and high‐fidelity coating for depositing ultra‐thin and conformal layers at the electrode surface to greatly improve the stability of various electrodes [2]. Besides electrochemical energy conversion and storage applications, ALD has widely been used in developing efficient electrode materials for solar cells and PEC cells by precisely depositing electrons and hole transport layers over semiconductor photoelectrodes [20, 21]. The emerging MLD and PEALD techniques have ability to deposit diverse materials on various supports for developing novel energy‐related catalysts. Currently, MLD and PEALD techniques offer development of advanced catalysts with high activity, stability, and selectivity by precise deposition of materials for energy conversion and storage applications energy [21].

### 4.1 Electrocatalysis

Hydrogen as clean energy has received tremendous attention in the recent time and renewable energy resource to be an alternative to fossil fuels. However, at the moment the main route of H_2_ generation is utilizing hydrocarbons. By contrast, water‐splitting through electrolysis could be an eco‐friendly and economical route to generate green H_2_. Developing water‐splitting technology relies on electrocatalysts. An electrocatalyst facilitates the acceleration of a particular electrochemical reaction. Thereby, during water‐splitting the electrical energy is stored in the form of chemical energy with the help of an electrocatalyst. HER and OER are two half‐cell reactions of water‐splitting. HER mechanism is described as a two‐step reaction of adsorption and desorption of H‐intermediates [168, 169, 170]. Typically, the electrochemical energy conversion and storage applications involve mainly the OER, HER, ORR, and hydrogen oxidation reaction (HOR) reactions [171, 172, 173, 174]. As mentioned in Section 3, most of the commercial catalysts are mainly precious metal‐based catalysts. Since a desirable electrocatalyst should satisfy high activity, cost effective and long‐term stability, decreasing noble metals content and keeping the activity high at the same time is a huge challenge for scientists [175, 176, 177, 178]. Regarding maximizing atomic utilization researchers did various studies on single‐atom catalysts especially on carbon‐based support materials (although carbon by itself does not show catalytic activity) like as Pt single‐atoms anchored on g‐C_3_N_4_ [143, 179, 180, 181, 182, 183, 184]. Moreover, in electrocatalysis, the interaction between the metal and the support, which is known as strong metal support interaction (SMSI), is highly important. SMIS usually is created between metals and metal oxide after high‐temperature annealing. Although SMIS could enhance the catalytic activity due to the electronic interaction between the metal and support, meanwhile, high‐temperature annealing brings some drawbacks of particle coarsening. It is noted that a weak bond between Pt and carbon support ends in low durability due to the decrease of Pt catalyst over time [183]. To prevent detachment, migration, and aggregation of catalysts nanocrystals during electrochemical reactions, conventional carbon support is often decorated with a thin metal oxide (MO) layer to induce SMSI [183]. Moreover, chemical interactions and electron transfer between MO and catalyst alter the electronic structure of the catalyst, leading improvement in catalytic performance [183]. It is worth mentioning that controlling the uniformity and thickness of MO layer on carbon support is the utmost important to avoid inferior charge transfer and catalytic activity. However, most of the conventional techniques such as wet‐chemical and chemical vapor deposition techniques have limitations in controlling both the size of catalysts and MO layer. Additionally, these techniques are incapable of precise tailoring of interfacial contact between catalysts and the MO layer. In this regard, ALD has great ability in precisely tailoring the interfacial properties of catalysts and inducing SMSI between catalysts and the MO layer by forming chemical bonds with active sites of the MO and metal catalysts [179]. For instance, Song et al. [183] recently reported a Pt‐WO_3_‐NCNT electrocatalyst where both the WO_3_ layer and Pt NPs were precisely deposited on N‐dopped carbon nanotube (NCNT) via two step ALD. The authors claimed that highly dispersed Pt NPs on WO_3_ interlayer ensured strong connection between Pt and WO_3_ which contributed to SMSI and enhanced electrochemical performance. The Pt‐WO_3_‐NCNT exhibited high durability and activity for ORR in comparison with Pt‐NCNT and commercial Pt/C catalysts, demonstrating the importance of metal oxide interface layer and ALD technique. Recently, ALD technique has been utilized to prepare various efficient electrocatalysts such as Pt/TiO_2_─C, Pt/MoO_3_‐CNT, and Pt/TiO_X_N_Y_/TiO_2_ and the enhanced electrochemical performance originated due to durability of the catalysts by SMSI [179, 183]. Therefore, the unique features of ALD can be used in depositing uniformly dispersed catalyst NPs with controllable size and tailoring the interface between support and catalysts [184]. In Table 4, information about some recently developed electrocatalysts prepared by ALD has been listed.

**TABLE 4:** List of some recent electrocatalysts for water‐splitting prepared by utilizing the ALD technique.

Electrocatalyst	ALD precursor//Reactant	Deposition temperature	Purpose	Ref.
Pd/Carbon nanofiber/Ni/NiO	Pd(hfac)_2_ **//**Formalin	220°C	OER	[185]
CoO_x_/FeO_x_/Carbon nanotube	FeCp_2_, (η_5_‐C_5_H_5_)_2_Fe**//**O_2_ CoCp_2_, (η_5_‐C_5_H_5_)_2_Co**//**O_2_	200°C^a^	OER	[186]
NiO_x_@Co_3_O_4_/Carbon cloth	(NiCp_2_, (η_5_‐C_5_H_5_)_2_Ni)**//**O_2_	260°C^a^	OER	[187]
(N,S)‐RGO@CoN	Co(^i^Pr_2_‐AMD)_2_ **//**Amine	300°C	OER	[188]
Cobalt phosphate/FTO	CoCp_2_ (TMP, (CH_3_O)_3_PO)**//**O_2_	300°C^a^	OER	[189]
NiO/FTO	Ni(MeCp)_2_ **//**O_2_ Ni(^t^Bu‐MeAMD)_2_ **//**H_2_O	300°C^a^ 150°C	OER	[190]
MoS_2_/3D printed Ti	MoCl_5_//H_2_S	350°C	HER	[191]
Pt@N‐Co_3_O_4_/Carbon cloth	MeCpPtMe_3_ **//**O_3_	280°C	HER	[192]
Pt nanocluster/TiO_2_NTs@3D‐Ti electrode	MeCpPtMe_3_ **//**O_2_	300°C	HER	[193]
FeP‐NCO@Nickel foam	Fe(CP)_2_ **//**O_3_	300°C	OER	[194]
Pd Single‐atom, NPs/Carbon	Pd(C_5_HF_6_O_2_)_2_ **//**Formalin	200°C	HER	[195]

^a^ Plasma.

In the following recent studies, the ALD technique has been utilized to develop efficient catalysts. For example, Ming Li et al. [196] employed ALD to carefully tune the morphology of the bimetallic Pt–Pd electrocatalyst. Due to the precise control of ALD on an atomic scale, it was possible to easily tune the structure of the Pt–Pd bimetallic catalyst by changing the deposition sequence. Pt encapsulated in Pd shell (Pt@Pd), Pd encapsulated in Pt shell (Pd@Pt), and Pt–Pd alloy was prepared as electrocatalysts to selectively reduce CO_2_ to formic acid. Pd@Pt only showed 7% faradic efficiency toward formic acid while it improved to 22% for Pt@Pd because the Pd shell exhibits higher selectivity toward formate in comparison with Pt shell. By contrast, Pt–Pd alloy, showed 46% (on average during 1 h) faradic efficiency toward formate. They observed that in the case of Pt–Pd, faradic efficiency increased during the first half hour and then decreased. They explained the mentioned observation by restructuring in the Pt–Pd which contributed to enhancing selectivity before deactivation. Moreover, Zhou et al. [196] reported ALD‐grown ultra‐thin amorphous molybdenum oxide (MoO_3_) layer into the ordered beaded‐like cobalt oxide (CoO) array on the three‐dimensional carbon cloth (CC) via ALD and applied the cowpea‐shaped MoO_3_@CoO/CC electrocatalyst in seawater splitting reactions. The authors claimed that the thin MoO_3_ layer effectively shielded chloride ions (Cl^−^) from reaching the catalyst's active interface and prevented chlorine evolution reaction (CER) while facilitated oxygen evaluation reaction (OER) in seawater. In another study, Yusufoglu et al. [196] reported ALD‐grown Cu_x_O overlayer on the surface of ZnO nanorods and applied the Cu_x_O/ZnO electrocatalyst for selective CO_2_ reduction to CO. The authors showed higher selectivity of the electrocatalyst with ALD‐grown Cu_x_O overlayer for CO_2_ reduction reaction compared to that of the bare ZnO. The enhanced selectivity of the Cu_x_O/ZnO electrocatalyst was ascribed to the uniform and conformal Cu_x_O overlayer on the ZnO surface. Recently, ALD has been used to mitigate the current gap in designing efficient energy‐related catalysts by considering their activity, stability and selectivity due to its ability of depositing ultra‐thin films and controlled NPs on complex geometry with high uniformity and atomic‐scale precision.

**FIGURE 12:**
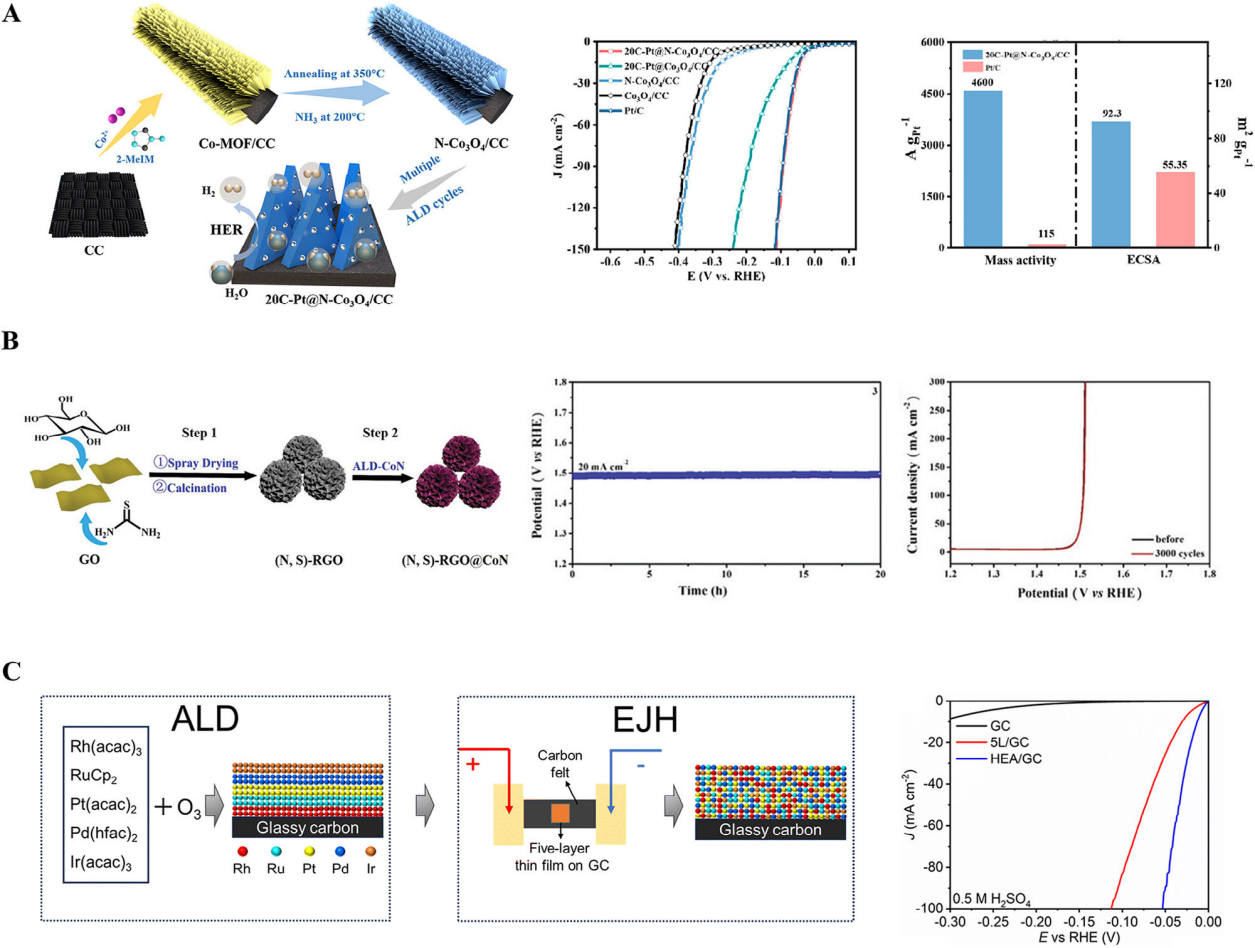
(A) Schematic of the preparation strategy of Pt@N‐Co_3_O_4_/CC catalyst and linear sweep voltammetry (LSV) towards HER for the Pt@N‐Co_3_O_4_/CC catalyst as well as comparison of mass activity and ECSA for Pt@N‐Co_3_O_4_/CC catalyst and Pt/C. Reproduced with permission [192]. Copyright 2024, American Chemical Society. (B) Schematic drawing for preparation for the S,N‐codoped RGO@CoN catalyst by a combination spray drying and ALD techniques, chronopotentiometry curve of the developed catalyst recorded at 20 mA cm^−2^, and LSV curve of the catalyst for OER measured before and after 3000 cycles of cyclic voltammetry. Reproduced with permission [188]. Copyright 2021, Wiley. (C) The schematic diagram for the preparation of RhRuPtPdIr high entropy alloy (HEA) on glassy carbon (GC) by ALD technique and the LSV curves show HER activity of the HEA/GC catalyst [197].

To carefully engineer the electronic metal support interaction (EMSI), Dung Yuan et al. [192]. employed ALD to deposit Pt particles on transition metal oxide support and evaluated electrochemical performance of the developed catalyst as shown in Figure 12A. The authors employed the ALD technique with its well‐known unique features to precisely tune the interface between the fine Pt particles and N‐doped Co_3_O_4_ (N‐Co_3_O_4_). To prepare N‐Co_3_O_4_/CC as support wet chemistry was utilized. Then Pt particles are deposited by ALD in certain cycle numbers. Conducting 20 cycles of ALD resulted in 0.5 wt% of Pt loading. However, EMSI regulation, 20C‐Pt@N‐Co_3_O_4_/CC had an overpotential of 34 mV at 10 mA cm^−2^ current density which was comparable with the performance of Pt/C (overpotential of 36 mV at 10 mA cm^−2^ current density). Moreover, at the overpotential of 50 mV, the mass activity of 4600 A g^−1^_Pt_ was calculated for 20C‐Pt@N‐Co_3_O_4_/CC while it was only 115 A g^−1^_Pt_ for commercial Pt/C. Such impressive performance came from optimizing the surface energy for adsorption/desorption of the intermediates by regulating the EMSI which became easily possible through the ALD technique. Moreover, Guo et al. [188] uniformly deposited CoN by the ALD technique on a sulfur and nitrogen (S,N) co‐doped reduced graphene oxide (RGO) support. Structural characterization of the developed catalyst confirmed the uniform deposition and strong chemical interaction of ALD‐deposited CoN with the S,N‐co‐doped RGO support which resulted in a current density of 10 mA cm^−2^ for OER at an overpotential of 220 mV. Also, the electrocatalyst showed good stability of 20 h at a current density of 20 mA cm^−2^ in a neutral electrolyte with a Faradic efficiency of nearly 100% (Figure 12B).

Zou et al. [197] take advantage of the particle‐like characteristic of the thin film deposited by ALD to prepare an electrocatalyst thin film with a large surface area. They successfully deposited uniformly five layers of Rh/Ru/Pt/Ir/Pd on a glassy carbon subsequently performed joule heating and measured electrocatalytic performance (Figure 12C). By applying electrical joule heating the 5‐layer thin film turned into a high entropy thin film composed of nanocrystalline structure and amorphous surface (known as high entropy metallic glass). At a current density of 10 mA cm^−2^, the high entropy thin film exhibited only 13 mV overpotential for HER in an acidic solution. The outstanding HER performance of the high entropy thin film was attributed to several reasons. Firstly, the ALD technique and surface amorphization created a ridge‐and‐valley surface which effectively increased the surface area. Moreover, the interaction between the electrocatalyst surface and hydrogen was accelerated by the modified electronic structure. The above‐mentioned studies suggest that the ALD technique precisely coat the complicated surfaces and also create various geometries by controlling the growth, precise control on composition and loading of the catalyst and enhancing the interaction between support and catalysts, resulted improvement in the performance of the electrocatalysts.

### 4.2 Photoelectrocatalysis

Utilization of wind and solar energies offers a sustainable way to net zero emissions [1]. However, the irregular nature of these energy sources due to geographical and seasonal variation demands new viable technologies for energy conversion and storage [198]. In this regard, PEC water‐splitting has received considerable attention for directly converting solar energy into storable chemical fuel by using a semiconductor photoelectrode and water. Solar light‐induced water‐splitting into hydrogen and oxygen on the TiO_2_ surface was first pioneered by Akira Fujishima and Kenichi Honda in 1972 since then various studies have been published on PEC water‐splitting reactions. Nevertheless, the energy conversion efficiency of this approach remains unsatisfactory so far due to several thermodynamic and kinetic limitations of water‐splitting reactions [198, 199]. The PEC water‐splitting is an endothermic process that requires around 1.23 eV (Δ*G*° = +237.2 KJ mol^−1^) energy for splitting water molecules into oxygen and hydrogen [198, 199, 200]. In this system, a semiconductor material absorbs solar light and generates photogenerated charge carriers such as electrons in the conduction band (CB) and holes in the valence band (VB). As a consequence, an electric field is generated in the semiconductor material which helps to separate the electrons and holes from each other spatially and facilitates to transfer of the holes at the semiconductor‐electrolyte interface for the oxidation reaction of water to oxygen whereas the electrons to the counter electrode for HER [1, 198, 199, 200].

Although this approach seems straightforward there are various aspects that restrain the efficiency of the PEC water‐splitting reactions. For example, TiO_2_ as a model n‐type semiconductor photoelectrode with bandgap energy 3.0–3.2 eV can absorb only the ultra‐violet portion (which is around 5% of the solar photon flux) and generate fewer amount of photogenerated charge carriers, resulting in low conversion efficiency. Therefore, bandgap modulation of the photoactive semiconductor material is required for extended absorption of the solar spectrum. However, the semiconductor material must have a higher bandgap energy of 1.23 eV which is the thermodynamic requirement of water‐splitting reactions. Moreover, the position of the VB maximum must be located more positive than the oxidation potential of water (1.23 V versus NHE), and the position of the CB minimum must be more negative than hydrogen reduction. Besides these thermodynamic requirements of the semiconductor material, there are many other kinetic aspects mainly related to light absorption, charge carrier generation, separation, transfer, and surface reaction that play a crucial role, together with some unwanted and inevitable processes like photocorrosion and charge carrier recombination or charge trapping. All these factors greatly influence the kinetics of water oxidation and reduction reactions [198, 199, 200].

**FIGURE 13:**
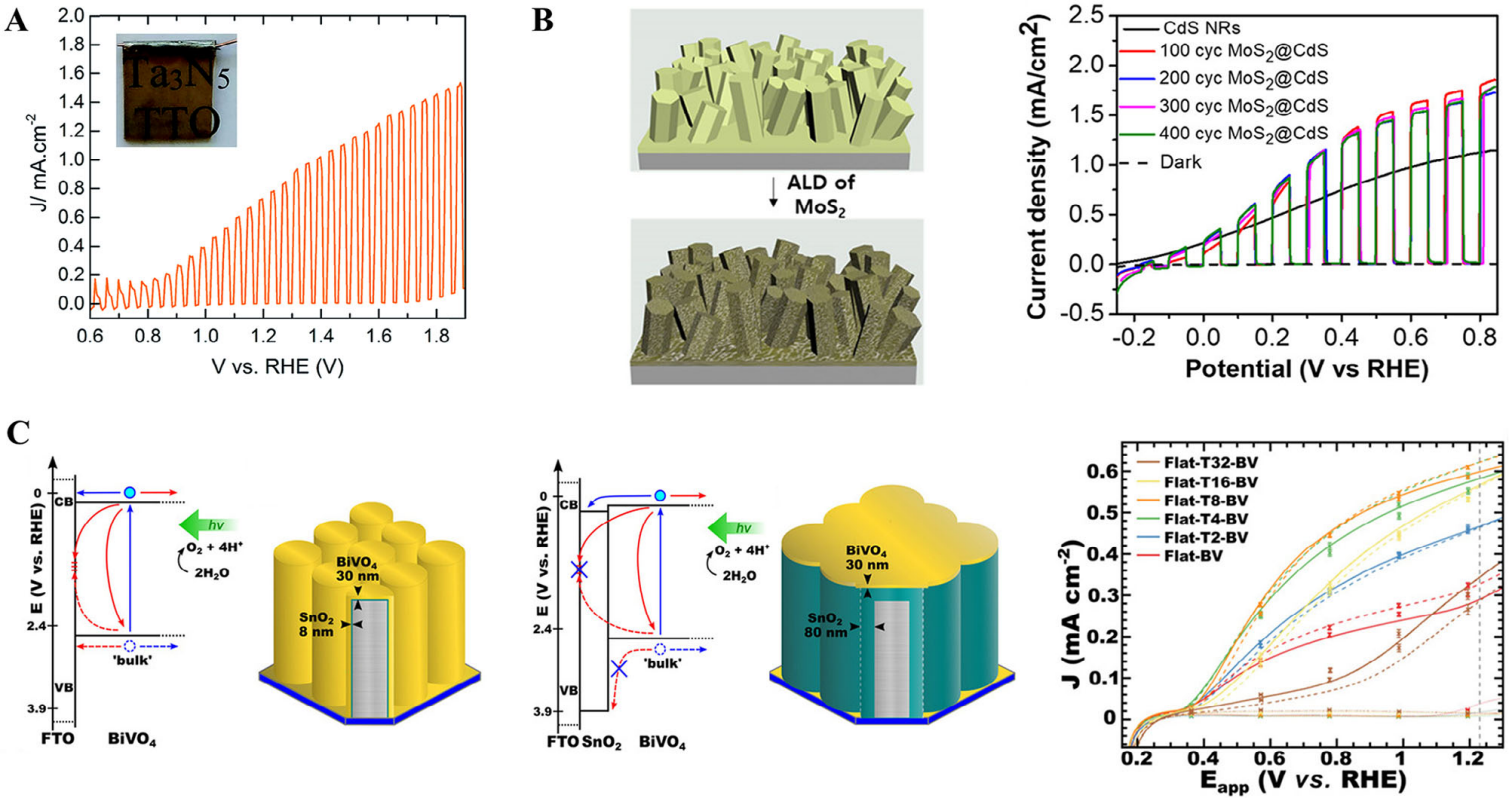
(A) LSV curve shows the light responsivity of the Ta_3_N_5_ film and the inset of (A) represents the digital photograph of the Ta_3_N_5_ film deposited on Ta‐doped TiO_2_ [202]. (B) schematic of CdS/MoS_2_ heterojunction photoanode and their LSV curves show improved PEC performance after ALD‐deposited MoS_2_ on CdS nanorod structures. Reproduced with permission [203]. Copyright 2019, American Chemical Society. (C) Schematics for charge transfer behavior of the FTO/BiVO_4_ and FTO/SnO_2_/BiVO_4_ photoanodes where BiVO_4_ as a light absorber on the FTO support was deposited by ALD and evaluated their PEC performance by LSV measurement. Reproduced with permission [204]. Copyright 2019, Wiley‐VCH Verlag GmbH & Co. KGaA, Weinheim.

Recently, the ALD technique has been used in surmounting both the above‐mentioned thermodynamic and kinetic limitations connected with the PEC water‐splitting reactions. Moreover, the capability of precisely controlled deposition and fine‐tuning of film thickness of ALD for various semiconductor materials has significantly improved the performance of the photoelectrodes for PEC water‐splitting [1, 198, 199, 200, 201, 202, 203, 204, 205, 206, 207, 208, 209, 210, 211, 212, 213, 214, 215, 216]. In addition, ALD has been effectively employed in synthesizing light‐absorbing materials (Table 5), underlayer coating (Table 6), overlayer coating (Table 7), interlayer coating (Table 8), and protective coatings (Table 9) for both photoanode and photocathode materials for efficient OER and HER [205, 206, 207, 208, 209, 210, 211, 212, 213, 214, 215, 216, 217, 218, 219, 220, 221, 222, 223, 224, 225, 226, 227, 228, 229, 230, 231, 232, 233, 234, 235, 236, 237, 238, 239, 240, 241, 242, 243, 244, 245, 246, 247, 248, 249, 250, 251]. For example, we published defective TiO_x_ thin film deposited by the ALD technique as a light‐absorbing material and comprehensively studied its PEC properties. We precisely controlled the film thickness and defect concentrations in the TiO_x_ film matrix by tuning the ALD deposition parameters. Also, we drew a correlation between the dependencies of defects on fundamental PEC properties [201]. Several other light‐absorbing materials have recently been deposited by the ALD technique to fabricate efficient photoelectrodes [202, 203, 204]. For example, Hajibabaei et al. [202] deposited a TaO_x_N_y_ (tantalum oxynitride) thin film as a light‐absorbing material by the ALD technique which was converted to tantalum nitride (Ta_3_N_5_) by ammonolysis. The light responsivity of the Ta_3_N_5_ film was checked by LSV measurement under light‐chopped conditions as shown in Figure 13A. Moreover, MoS_2_ on CdS nanostructure and BiVO_4_ on Sb‐doped SnO_2_ nanotube arrays were deposited as light absorbers by the ALD to construct heterojunction photoanodes and improve their PEC performance (Figure 13B) [203]. In addition, photogenerated charge transfer behavior of the FTO/BiVO_4_ and FTO/SnO_2_/BiVO_4_ photoanodes where BiVO_4_ as a light absorber on the FTO support was deposited by ALD and evaluated their PEC performance by LSV measurement (Figure 13C).

**TABLE 5:** Various photoactive materials are deposited using the ALD technique to fabricate efficient photoelectrodes for PEC water‐splitting reactions.

Photoelectrode	Light absorber	ALD precursor//Reactant	Deposition temperature	Purpose	Ref.
Si/Hf:ZnO/C/Pt	Hf:ZnO	Zn(C_2_H_5_)_2_, Hf(EtMeN)_4_//H_2_O	250°C	OER	[205]
TaO_x_N_y_	TaO_x_N_y_	Ta(NMe_2_)_5_//H_2_O	90°C	OER	[202]
TiO_2_/ZnO	ZnO	Zn(C_2_H_5_)_2_//H_2_O	250°C	OER	[206]
Porous anodic Al_2_O_3_/Au/Fe_2_O_3_	α‐Fe_2_O_3_	Fe(Cp)_2_//O_3_	200°C	OER	[207]
FTO/SnS_2_	SnS_2_	[(CH_3_)_2_N]_4_Sn//10.4% H_2_S/N_2_	140°C	OER	[208]
CdS/MoS_2_	MoS_2_	MoCl_5_//4% H_2_S/Ar	250°C	OER	[203]
ZnO/SnO_2_/BiVO_4_	BiVO_4_	VO(OC^i^Pr_2_)_3_, Bi(OCMe_2_^i^Pr)_3_//H_2_O	150°C	OER	[209]
TiO_2_/SnO_2_/BiVO_4_	BiVO_4_	VO(OC^i^Pr_2_)_3_, BiPh_3_//H_2_O, MeOH	130°C	OER	[204]
Al:TiO_2_	Al:TiO_2_	TiCl_4_, Al(CH_3_)_3_//H_2_O	100°C	OER	[210]
Stainless steel/TiO_2_	TiO_2_	TiCl_4_//H_2_O	300°C	OER	[211]
ZnO/TiO_2_	ZnO	Zn(C_2_H_5_)_2_//O_3_	160–240°C	OER	[212]
TiO_x_/FTO	TiO_x_	[(CH_3_)_2_N]_4_Ti//H_2_O	200°C	OER	[201]

**TABLE 6:** Various underlayer coatings are deposited using the ALD technique to suppress charge recombination in the photoelectrodes for PEC water‐splitting reactions.

Photoelectrode	Underlayer	ALD precursor//Reactant	Deposition temperature	Purpose	Ref.
SnO_2_/Fe_2_O_3_	SnO_2_, 2 nm	Sn(NMe_2_)_4_//H_2_O	230°C	OER	[215]
SnO_2_/BiVO_4_	SnO_2_, 8 nm	SnCl_4_//H_2_O	300°C	OER	[213]
WO_3_/Fe_2_O_3_	WO_3_, 2 nm	(t‐Bu_2_N)_2_W(NMe_2_)_2_//H_2_O	260°C	OER	[215]
Nb_2_O_3_/Fe_2_O_3_	Nb_2_O_3_, 2 nm	Nb(OCH_2_CH_3_)_5_//H_2_O	200°C	OER	[215]
Ga_2_O_3_/Fe_2_O_3_	Ga_2_O_3_, 2 nm	Ga_2_(NMe_2_)_6_//H_2_O	150°C	OER	[215]
TiO_2_/Ti:Fe_2_O_3_	TiO_2_	Ti(NMe_2_)_4_//H_2_O	150°C	OER	[216]
Ta:TiO_2_/Ta_3_N_5_/Co─Pi	Ta:TiO_2_ 0–5%, Ta 100 nm	Ti(O^i^Pr)_4_, Ta(NMe_2_)_5_//H_2_O	250°C	OER	[202]
TiO_2_/Fe_2_O_3_	TiO_2_, 0.2–2.5 nm	Ti(NMe_2_)_4_//O_2_	80°C	OER	[217]
TiO_2_/Fe_2_O_3_/Ni(OH)_2_	TiO_2_	Ti(NMe_2_)_4_//H_2_O	170°C	OER	[218]

**TABLE 7:** Various overlayer coatings are deposited using the ALD technique to passivate the surface states of the photoelectrodes for PEC water‐splitting reactions.

Photoelectrode	Overlayer	ALD precursor//Reactant	Deposition temperature	Purpose	Ref.
TiO_2_/Al_2_O_3_	Al_2_O_3_, 1–20 nm	Al(Me)_3_//H_2_O	100–400°C	OER	[219]
α‐Fe_2_O_3_/Al_2_O_3_	Al_2_O_3_, 0.1–2 nm	Al(Me)_3_//H_2_O	200°C	OER	[221]
Ag@TiO_2_/Al_2_O_3_	Al_2_O_3_, ∼2 nm	Al(Me)_3_//H_2_O	200°C	OER	[223]
WO_3_/Al_2_O_3_	Al_2_O3, ∼5 nm	Al(Me)_3_//O_2_ ^a^	100°C	OER	[222]
CdS+PbS@ TiO_2_/Al_2_O_3_	Al_2_O_3_, 1.5 nm	Al(Me)_3_//H_2_O	150°C	OER	[224]
WO_3_/BiVO_4_/ZnO	ZnO, ∼15 nm	Zn(C_2_H_5_)_2_//H_2_O	120°C	OER	[225]
Nanowires TiO_2_/TiO_2_	TiO_2_, 10 nm	Ti(O^i^Pr)_4_//H_2_O	250°C	OER	[226]

^a^ Plasma.

**TABLE 8:** Various interlayer coatings are deposited using the ALD technique to facilitate charge transfer in the photoelectrodes for PEC water‐splitting reactions.

Photoelectrode	Interlayer	ALD precursor//Reactant	Deposition temperature	Purpose	Ref.
n‐Si/CoO_x_/NiO_x_	CoO_x_, 2–3 nm	Co(Cp)_2_//O_3_	150°C	OER	[230]
CuO/ZnO/TiO_2_	ZnO	Zn(C_2_H_5_)_2_//H_2_O	200°C	HER	[227]
Cu_2_O/Al:ZnO/TiO_2_/Pt	Al:ZnO	Zn(C_2_H_5_)_2_, Al(Me)_3_//H_2_O	120°C	HER	[74]
n‐Si/SiO_x_/CoO_x_/NiO_x_	SiO_x_, 2 nm	Co(Cp)_2_//O_3_	150°C	OER	[230]
Cu_2_O/ZnO/Al_2_O_3_/TiO_2_/Pt	4 nm ZnO, 0.17 nm Al_2_O_3_, and 11 nm TiO_2_	Zn(C_2_H_5_)_2_//H_2_O, Al(Me)_3_//H_2_O, and Ti(O^i^Pr)_4_//H_2_O	200°C	HER	[231]
ZnO/SnO_2_/BiVO_4_	SnO_2_, 8 nm	Sn(NMe_2_)_4_//O_3_	115°C	OER	[204]
CuGeSe/CdS/TiO_2_/MoS_2_	TiO_2_, 5 nm	[(CH_3_)_2_N]_4_Ti//H_2_O	150°C	HER	[228]
Si/TiO_2_/ZnO	TiO_2_	Ti(O^i^Pr)_4_//H_2_O	200°C	OER	[232]
n^+^p^−^Si/Al_2_O_3_/Pt	Al_2_O_3_, 2.3 nm	Al(CH_3_)_3_//H_2_O	230°C	HER	[229]
n^+^p^−^Si/Al_2_O_3_/Pt	Al_2_O_3_ 4.5 nm	Al(CH_3_)_3_//H_2_O	200°C	HER	[233]
n‐Si/Al_2_O_3_/NiO_x_	Al_2_O_3_, 2.6 nm	Al(CH_3_)_3_//H_2_O	230°C	OER	[234]

**TABLE 9:** Various protective coatings are deposited using the ALD technique to prevent photocorrosion of the photoelectrodes for PEC water‐splitting reactions.

Photoelectrode	Protective layer	ALD precursor//Reactant	Deposition temperature	Purpose	Ref.
Mo:BiVO_4_/Nb:TiO_2_/Fe:NiO	Nb:TiO_2_, ∼2.5 nm	Ti(O^i^Pr)_4_, Nb(OEt)_5_//H_2_O	200°C	OER	[236]
WO_3_/BiVO_4_/TiO_2_	TiO_2_, ∼2 nm	Ti(NMe_2_)_4_//H_2_O	200°C	OER	[235]
Nanoporous p^+^Si/HfO_2_	HfO_2_ 1–6 nm	Hf(NMe_2_)_5_//H_2_O	95°C	HER	[237]
n^–^Si/ZrO_2_/NiFe	ZrO_2_ 1–3 nm	Zr(N(EtMe))_5_//O_2_	200°C	OER	[238]
p^+^Si/MnO_x_	MnO_x_ 10 nm	(Et_2_Cp)_2_Mn//H_2_O	150–200°C	OER	[239]
p^+^n^–^Si/NiO_x_	NiO_x_ 50 nm	Ni(Cp)_2_//O_3_	100°C	OER	[240]
Nanorods ZnO/Ta_2_O_5_	Ta_2_O_5_ 1.5 nm	Ta(NMe_2_)_5_//H_2_O	200°C	OER	[241]
TiO_2_/CdS/ZnO	ZnO ∼7 nm	Zn(C_2_H_5_)_2_//H_2_O	145°C	OER	[242]
n^–^p^+^Si/SiO_2_/Al_2_O_3_/Pt	Al_2_O_3_ 2.3 nm	Al(Me)_3_//H_2_O	230°C	HER	[229]
p‐Si/TiO_2_	TiO_2_ 4 nm	Ti(O^i^Pr)_4_//H_2_O	250°C	HER	[250]
Cu_2_O/TiO_2_	TiO_2_ 45 nm	Ti(NMe_2_)_4_//H_2_O	80–150°C	HER	[243]
TaO_x_N_y_/Ta:TiO_2_/IrO_2_	Ta:TiO_2_ (1.6% Ta)	Ti(O^i^Pr)_4_, Ta(NMe_2_)_5_//H_2_O	200–500°C	OER	[202]
CuO/ZnO/TiO_2_	TiO_2_ 4–143 nm	Ti(O^i^Pr)_4_//H_2_O	200°C	HER	[227]
PEDOT:PS/MAPbI_3_/TiO_2_/Pt	TiO_2_ 18–40 nm	Ti(NMe_2_)_4_//H_2_O	120°C	HER	[244]
Cu_2_O/Al:ZnO/TiO_2_/Pt	TiO_2_ 11 nm	Ti(O^i^Pr)_4_//H_2_O	200°C	HER	[245]
NiMo/GaAs/InGaP/TiO_2_/Ni	TiO_2_ 150 nm	Ti(NMe_2_)_4_//H_2_O	150°C	OER	[246]
Mo/Cu_2_ZnSnS_4_/CdS/Al:ZnO/TiO_2_/Pt	TiO_2_ 1–6 nm	Ti(NMe_2_)_4_//H_2_O	150°C	HER	[247]
CdTe/TiO_2_/NiO_x_	TiO_2_ 140 nm	Ti(NMe_2_)_4_//H_2_O	150°C	OER	[248]
Cu_2_O/Ga_2_O_3_/TiO_2_/Pt	TiO_2_ 15 nm	Ti(NMe_2_)_4_//H_2_O	120–160°C	HER	[249]
p‐GaAs/TiO_2_/Pt	TiO_2_ 6 nm	TiCl_4_//H_2_O	150–300°C	HER	[250]
n^−^p^+^Si/TiO_2_/NiCrO_x_	TiO_2_ 94 nm	Ti(NMe_2_)_4_//H_2_O	150°C	OER	[251]

**FIGURE 14:**
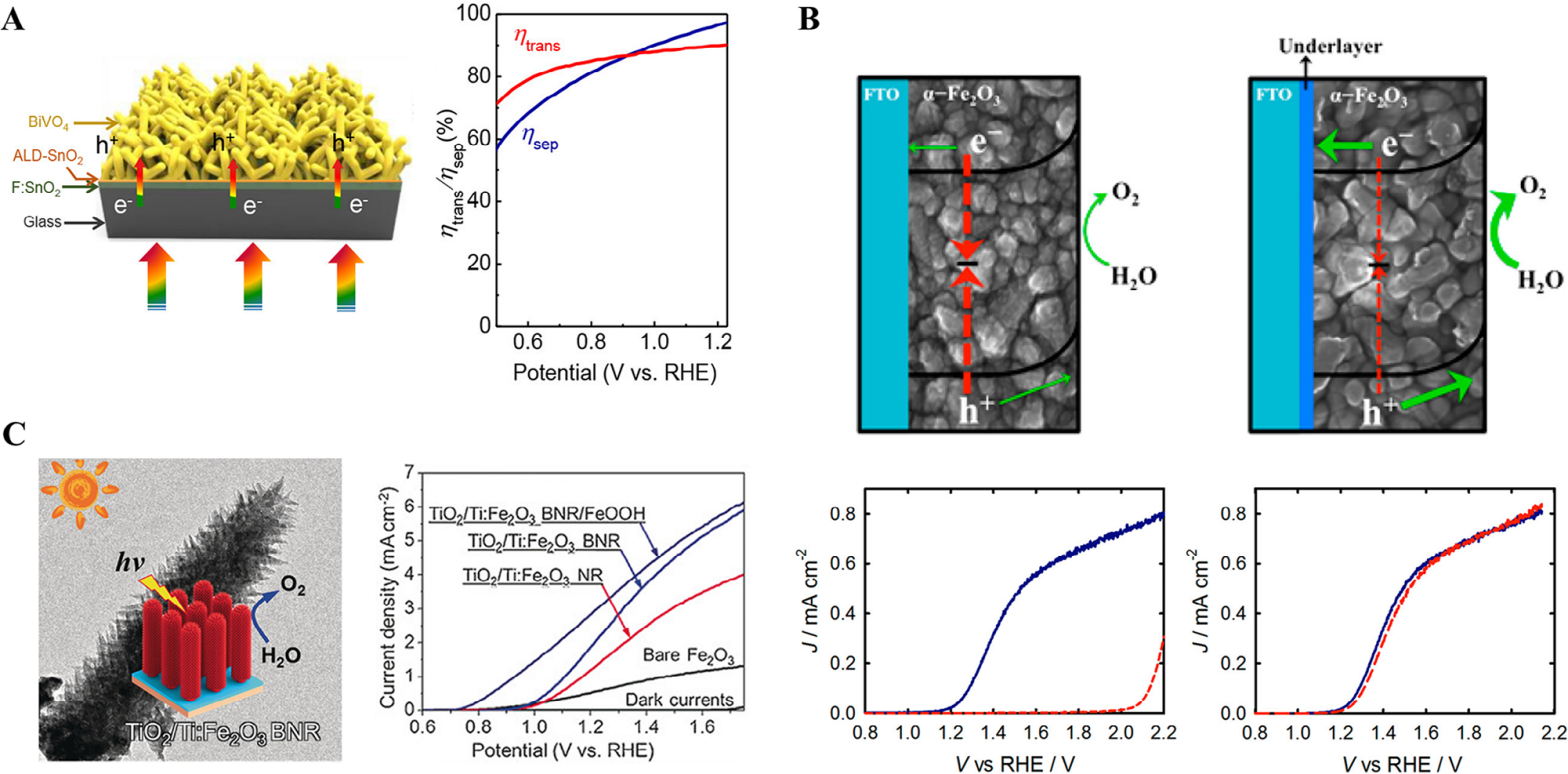
(A) represents the schematic of an ALD‐SnO_2_ underlayer coated BiVO_4_ photoanode on an FTO substrate with the curves showing photogenerated charge separation and transfer efficiencies versus potential. Reproduced with permission [214]. Copyright 2021, American Chemical Society. (B) shows a schematic drawing of photoanodes with a ∼2 nm thick Ga_2_O_3_ underlayer at the substrate‐hematite interface and the LSV curves exhibit improved PEC performance after the thin Ga_2_O_3_ underlayer coating. Reproduced with permission [215]. Copyright 2014, American Chemical Society. (C) shows a TEM image and schematic of a nanostructured hematite photoanode with a thin titanium dioxide layer coated at the substrate‐hematite interface. The LSV curves in (C) show the improved PEC performance of the TiO_2_ underlayer‐coated photoanode. Reproduced with permission [216]. Copyright 2017, Wiley‐VCH Verlag GmbH & Co. KGaA, Weinheim.

Besides light absorbing materials deposition, the ALD technique has also been used for underlayer coating to prevent interfacial charge recombination and facilitate charge transfer. Moreover, this technique can be used for uniform seed layer deposition to synthesize the controlled orientation of nanostructures. For instance, the controlled and oriented rutile SnO_2_ nanowires (NWs) as photoanode material on various conducting substrates were synthesized by ALD seeding and the experimental results showed that the seed layer growth controls the nucleation of the SnO_2_ NWs. Moreover, the length, width, and density of the SnO_2_ NWs were controlled by tuning the thickness and crystallographic properties of the seed layer [213]. The ALD can be successfully used for precise modulation of the interface of the photoelectrodes. For instance, our group reported a nanoporous BiVO_4_ photoanode with improved PEC water‐splitting performance. In this work, a conformal and ultrathin SnO_2_ underlayer was deposited by ALD at the interface of conductive fluorine‐doped tin oxide (FTO)‐coated glass substrate and BiVO_4_ to block the defect density and reduce the interfacial resistance (Figure 14A). Typically, the FTO layer contains defects and some defect sites act as charge recombination centers or charge trapping sites which results in an accumulation of electrons at the FTO/BiVO_4_ interface, allowing the photogenerated holes to reach the interface and recombine with the trapped electrons, resulting in reduction in PEC performance. However, the thin ALD‐SnO_2_ layer prevented the photogenerated holes from recombining with the electrons at the FTO/BiVO_4_ interface, resulting in fewer recombination and higher charge collection efficiency [214]. Similarly, a thin Ga_2_O_3_ layer (Figure 14B) and TiO_2_ (Figure 14C) layer were deposited through the ALD technique at the FTO substrate‐hematite interface by Zandi et al. [215] and Luo et al. [216], respectively to improve the PEC performance by suppressing interfacial charge recombination.

Many papers have been published on the successful deposition of underlayer coating in photoelectrodes for PEC water‐splitting applications (Table 6).

**FIGURE 15:**
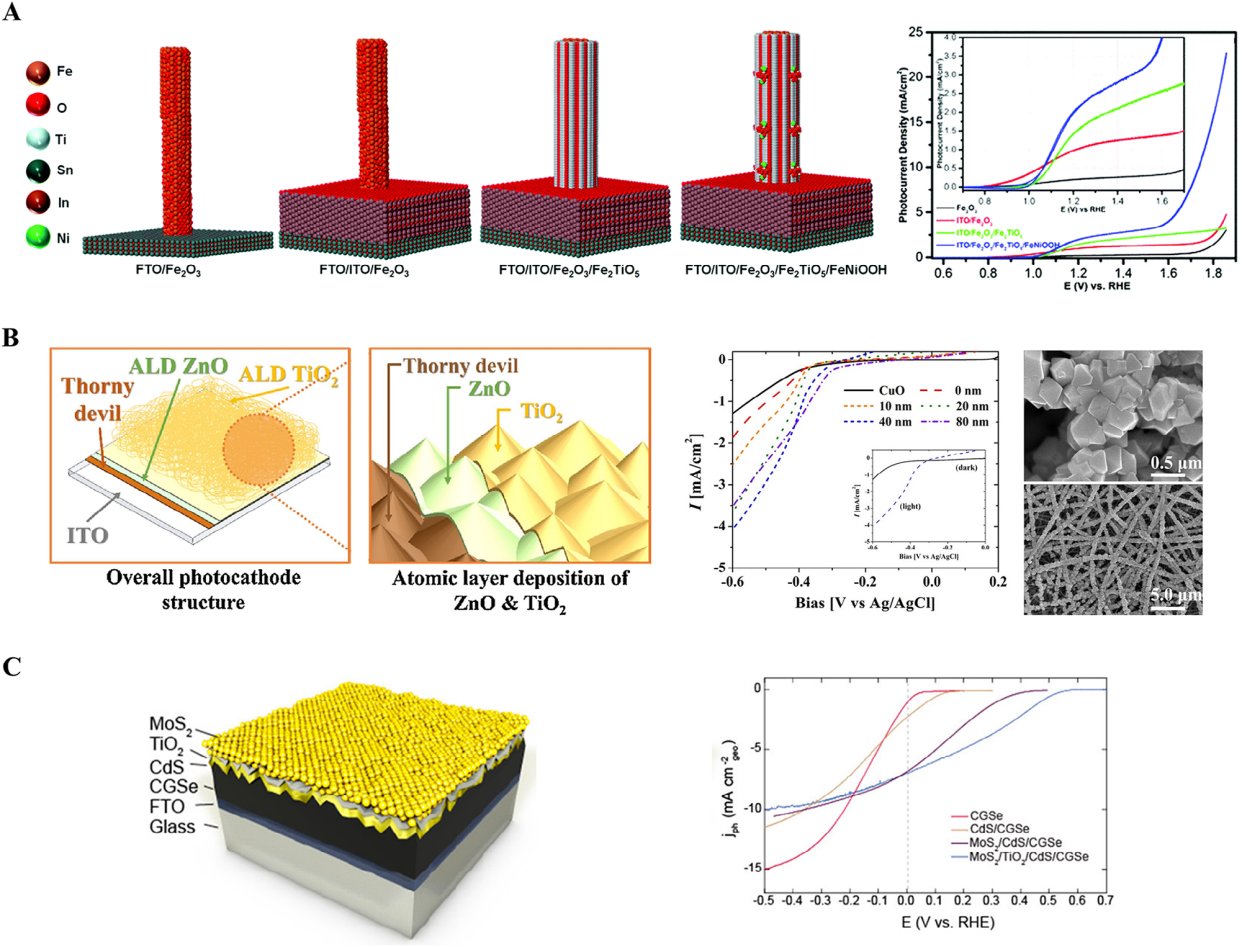
(A) represents the schematic drawing of surface and interface‐engineered hematite photoanodes by the ALD technique with their corresponding photocurrent density measured by LSV. Reproduced with permission [220]. Copyright 2017, The Royal Society of Chemistry. (B) shows the schematic of nanostructured CuO photocathode with thin ZnO and TiO_2_ layers by the ALD, the LSV curves for their PEC performance, and the scanning electron microscope (SEM) images of the photocathode. Reproduced with permission [228]. Copyright 2017, Elsevier. (C) represents the schematic and LSV curves of the MoS_2_/TiO_2_/CdS/CGSe heterojunction photocathode where TiO_2_ was used as a thin interfacial layer. The LVS curve shows that the photocurrent onset for the photocathode shifted to a more positive value after the TiO_2_ interfacial layer coating. Reproduced with permission [229]. Copyright 2019, American Chemical Society.

As discussed earlier, the PEC water‐splitting reactions involve many complicated processes at the electrode‐electrolyte interface that diminish PEC activity. ALD allows the deposition of ultrathin overlayer coatings (Table 7) that address the limitations of surface charge recombination and help to passivate surface states [219]. For instance, Tang et al. [220] reported a surface/interface‐engineered hematite (α‐Fe_2_O_3_) nanorod‐shaped photoanode for PEC water oxidation reaction by the ALD. In this work, a thin indium tin oxide (ITO) layer was deposited at the interface of FTO/α‐Fe_2_O_3_ to reduce interfacial resistance and smooth photogenerated electron transfer through the FTO back contact. An ultra‐thin and conformal TiO_2_ layer was deposited over the α‐Fe_2_O_3_ nanorod and subsequently heat‐treated at 750°C to convert the surface TiO_2_ layer into a Fe_2_TiO_5_ layer (Figure 15A). The resultant ITO/Fe_2_O_3_/Fe_2_TiO_5_ photoanode displayed a photocurrent of more than 10 times higher compared to the pristine Fe_2_O_3_ nanowire‐shaped photoanode (from 0.205 to 2.2 mA cm^−2^ at 1.23 V versus RHE and 1 Sun). Moreover, Formal et al. [221] reported a thin Al_2_O_3_ layer deposited by ALD on nanostructured Fe_2_O_3_ to passivate surface states. It was shown that the overlayer coatings reduced the number of electrons trapping sites on the surface of the photoelectrodes, resulting in facile photoelectron transfer. At the same time, it consumes more holes at the photoelectrode surface, suppressing the recombination process, and facilitating water oxidation [222]. Several overlayer coatings on photoelectrodes by ALD have been published as shown in Table 7.

Typically, in the PEC water‐splitting, a single semiconductor photoelectrode cannot fulfill all the tasks like light absorption, charge carrier generation, charge separation and transfer, and reactions at the semiconductor‐electrode interface simultaneously. Therefore, an additional semiconductor with a favorable band energy alignment or a catalyst is required to improve the overall PEC performance. However, different junctions such as the Schottky junction and semiconductor‐semiconductor junction are formed at the light‐absorbing semiconductors and catalysts interface that may cause charge transfer resistance [198]. In such cases, an interlayer between the semiconductors and catalysts can facilitate the charge transfer at the junction, resulting in the enhancement of the PEC performance [227]. In this regard, ALD has been extensively used to successfully deposit various interlayers in the photoelectrodes for PEC water‐splitting reactions (Table 8). For instance, Kim et al. [228] fabricated a nanostructure CuO photocathode and then coated it with thin ZnO and TiO_2_ layers by the ALD to improve the electron transfer property and prevent the CuO photocathode from corrosion (Figure 15B). Moreover, Hellstern et al. [229]. reported MoS_2_/TiO_2_/CdS/CGSe heterojunction photocathode where TiO_2_ was used as a thin interfacial layer to facilitate interfacial charge transfer that led to the positive shift of onset potential as shown in Figure 15C.

**FIGURE 16:**
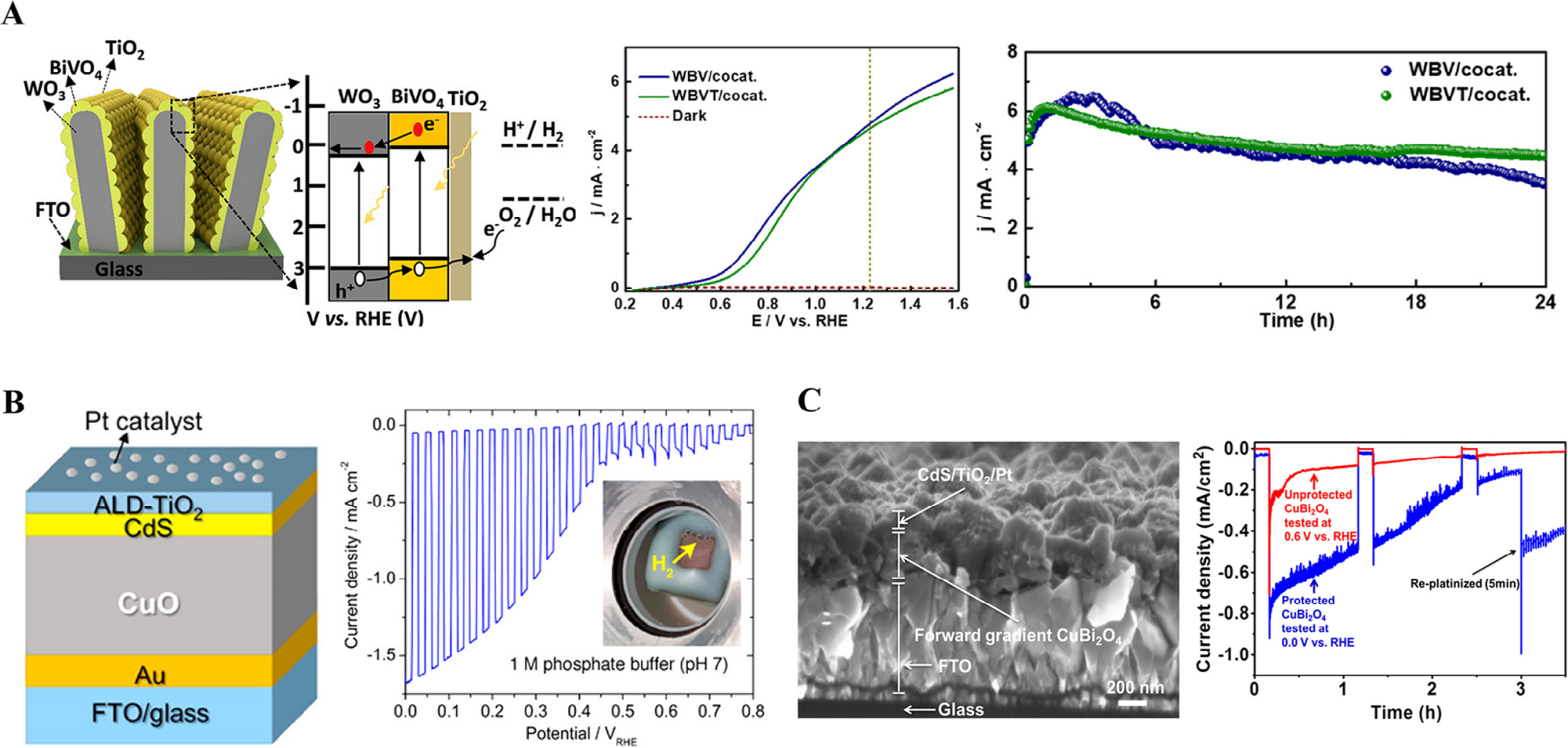
(A) represents the schematic drawing of the WO_3_/BiVO_4_ heterojunction photoanode with a thin TiO_2_ protective layer and its charge transfer behavior. Moreover, (A) shows the LSV and stability (photocurrent versus time) curves of the photoanodes with/without the ALD‐TiO_2_ protective layer. Reproduced with permission [235]. Copyright 2022, American Chemical Society. (B) represents the schematic of a CuO/CdS heterojunction photoelectrode with a thin ALD‐TiO_2_ protective layer and an LSV curve to show the photocurrent density and photoresponsivity of the photocathode. The inset image in the LSV curve represents the evolution of H_2_ gas. Reproduced with permission [252]. Copyright 2017, American Chemical Society. (C) exhibits the cross‐sectional SEM image of the CuBi_2_O_4_/CdS/TiO_2_/Pt photocathode where the ALD‐TiO_2_ acts as a protective layer and the photostability (photocurrent versus time) curve of the photoelectrode. Reproduced with permission [253]. Copyright 2017, American Chemical Society.

Apart from the nanostructure modulation and interface engineering of the photoanodes by the ALD, preventing photocorrosion and dissolution of the photoelectrodes under harsh PEC conditions remains a major concern. In this regard, ALD is widely used to deposit protective layers over the photoelectrodes to prevent their photocorrosion and dissolution (Table 9) [235, 236, 237, 238, 239, 240, 241, 242]. By taking advantage of the ALD technique, we reported a nanostructured WO_3_/BiVO_4_ heterojunction photoanode with a conformal and ultra‐thin (∼2 nm) TiO_2_ protective layer on top of the photoanode. The experimental results confirm that the thin TiO_2_ layer did not affect the photogenerated hole transport at the photoelectrode‐electrolyte interface and efficiently protected the photoanode from photocorrosion, resulting in sustained water oxidation performance (Figure 16A). However, a thicker TiO_2_ protective layer (∼5 nm) on the photoanode significantly reduced the PEC performance of the sample owing to the suppressed hole transportation at the photoelectrode–electrolyte, signifying the appropriateness of the ALD technique in designing efficient photoanodes [235].

Moreover, Septina et al. [252] reported a thin ALD‐TiO_2_ layer on a CuO/CdS heterojunction photocathode for stable solar hydrogen generation (Figure 16B) and Wang et al. [253] reported the ALD‐TiO_2_ layer‐protected CuBi_2_O_4_ photocathode and it prevented photocorrosion, resulting in improved stability (Figure 16C).

Besides PEC water‐splitting reactions, the ALD technique has broadly been used to design catalysts for photocatalytic reactions [254, 255, 256]. The catalysts for the photocatalytic reactions need suitable support materials and good catalyst–support interaction. Moreover, homogeneous deposition of catalyst materials over support significantly affects the catalytic performance. Different ALD‐based nanostructured catalysts such as inverse opal scaffolds and coke‐shell structures have been reported for environmental remediation and CO_2_ reduction [254]. Moreover, the electronic states of the ALD catalysts can be tuned by changing the chemical properties of the substrate. For example, Liu et al. [255] reported a Cu catalyst on pristine‐TiO_2_ and black‐TiO_2_ substrates by ALD technique for CO_2_ reduction to CO and CH_4_ generation. The experimental results showed that the pristine‐TiO_2_@Cu outperformed the black‐TiO_2_@Cu catalysts for the photocatalytic CO_2_ reduction. Detailed analyses revealed that the Cu^0/+^ electronic states remained predominant in the pristine‐TiO_2_ substrate whereas the Cu^2+^ state remained as major in the black‐TiO_2_, implying the electronic state of the ALD‐Cu is dependent on the chemical properties of the substrate. Most of the ALD‐deposited photocatalysts were synthesized to exploit morphology modification for boosting and controlling surface reaction kinetics [257]. Furthermore, this technique can be broadened to synthesize new materials and architectures to achieve improved photocatalytic reactions.

### 4.3 Solar Cells

Photovoltaic cells or solar cells, commercially mature technologies, are electronic devices that convert light energy directly into electrical energy and ALD is inextricably involved with these technologies [258]. In solar cell technologies, ALD becomes an essential part and the materials deposited by the ALD have important roles in photon absorption, charge carrier generation, separation, transport, surface/interface passivation, and encapsulation layer for device stability [259]. Recently, rapid progress in commercializing solar cell technologies has been facilitated by the ALD owing to its competency to deposit precise, compact, and high‐fidelity ultrathin films over a large area. Typically, surface and bulk defects of semiconductor materials help to recombine the charge carriers that greatly affect the efficiency of the solar cells [258, 259]. The ALD is successfully implemented to passivate the surface defect states of the semiconductors in solar cell technologies. For instance, various materials such as Al_2_O_3_, SiO_2_, TiO_2_, and HfO_2_ have recently been used for surface passivation of crystalline silicon (c‐Si) solar cells and have shown excellent levels of surface passivation with improved cell efficiency [260]. In c‐Si solar cells, 5 nm ALD Al_2_O_3_ passivation layers are effectively implemented at a large fraction of the passivation emitter rear contact (PERC) solar cell. Utilization of the ALD‐Al_2_O_3_ layer in the PERC solar cells that are commercially available validates the capability of the ALD technique in future solar cell industries. Moreover, various transition metal oxides (TMOs) are used as the contact layers for electron‐selective and hole‐selective in the c‐Si solar cells. The TMOs with suitable band energy positions concerning Si can be used as contact passivation layers. For potential electron‐selective contacts TiO_2_, ZrO_2_, and Ta_2_O_5_, are used whereas WO_3_, NiO_x_, V_2_O_5_, and MoO_3_, are used as the hole‐selective contact layers in the c‐Si solar cell [261, 262, 263, 264, 265, 266].

**FIGURE 17:**
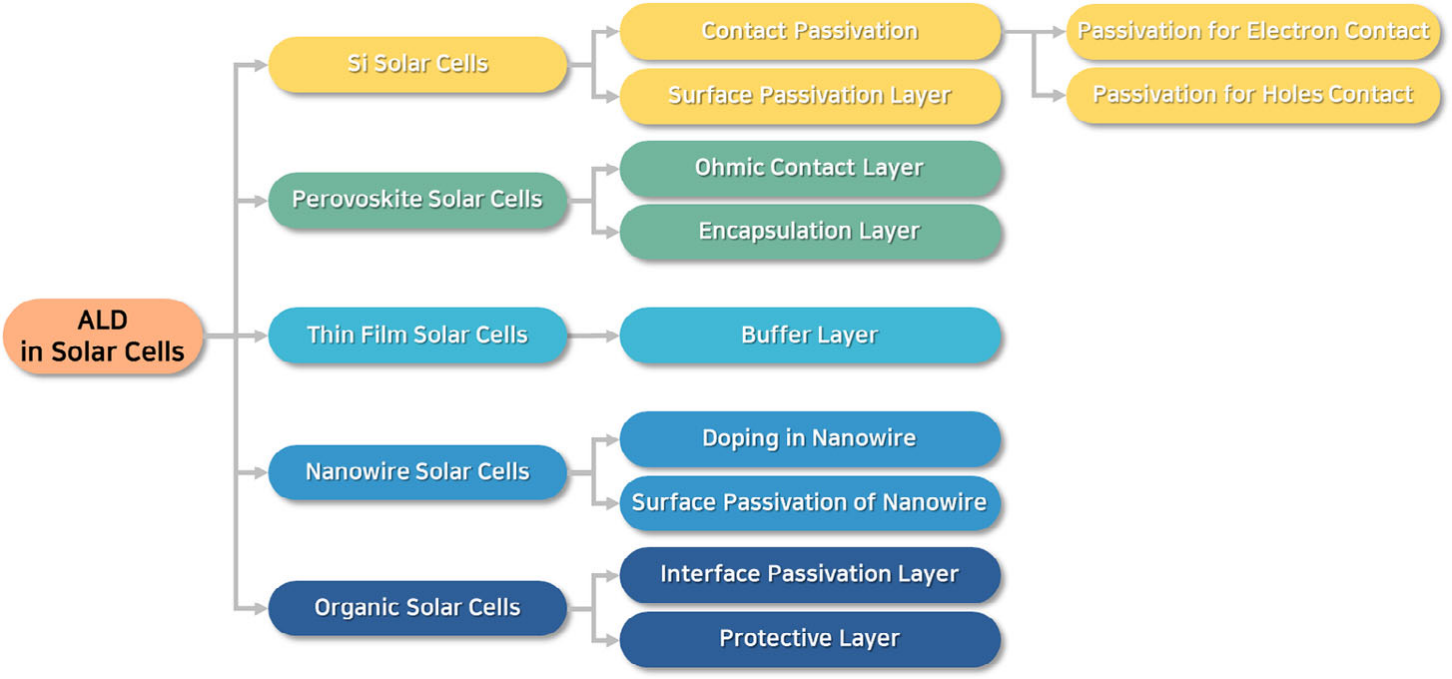
A flow chart diagram shows the utilization of ALD in depositing different layers in solar cell technology.

Besides c‐Si solar cells, ALD has been successfully used in high‐efficiency hybrid perovskite solar cells (PSCs). It is used to deposit an ultrathin Al_2_O_3_ layer on mesoscopic SnO_2_ or TiO_2_ layers for suppressing surface defects and improving the stability of the PSCs [267]. For the fabrication of efficient perovskite/Si two‐terminal tandem cells, the ALD‐TiO_2_ film has been used as a good ohmic contact at TiO_2_/p^+^‐Si [267, 268]. Moreover, ALD has been implemented in depositing compact encapsulation layers (SnO_2_ or Al_2_O_3_ layers) that protect PSCs from volatilization of the organic layer, humidity, temperature, and atmospheric oxygen, leading to improved stability and efficiency of the cells [267]. ALD has also been used to deposit different light‐absorbing materials such as PbS, CdS, Cu_2_S, Sb_2_S_3_, Bi_2_S_3_, In_2_S_3_, SnS, CuInS_2_, CuSbS_2_, CuInSe_2_, Cu(In, Ga)(S, Se)_2_, Cu_2_ZnSn(S, Se)_4_, and Cu(In, Ga)Se_2_ in solar cells [269]. Recently, conformal and ultrathin metal oxide layers have been deposited in organic, nanowire, and thin‐film solar cells for their performance improvement. Utilization of ALD in different solar cells for precise and controlled deposition of various materials has been shown in Figure 17.

### 4.4 Fuel Cells

Fuel cells (FCs) are devices where chemical energy is converted into electrical energy with the help of electrochemical reactions in an eco‐friendly manner [270]. Recently, the FCs have emerged as a promising, reliable, and net‐zero emissions technology. Different fuel cells such as proton exchange membrane fuel cells (PEMFCs), anion exchange membrane fuel cells (AEMFCs), phosphoric acid fuel cells (PAFCs), molten carbonate fuel cells (MCFCs), solid oxide fuel cells (SOFCs), and protonic ceramic fuel cells (PCFCs) have been explored so far [271, 272, 273, 274]. Typically, the FCs are composed of an ionically conductive electrolyte material sandwiched between an anode and a cathode, and oxygen and hydrogen are commonly used as fuels for ORR and HOR, respectively to produce water and electricity [271, 272, 273, 274]. Moreover, the anode and cathodes are coated with catalysts to facilitate the electrochemical reactions and produce electric energy.

The catalysts for FCs, particularly for PEMFCs which are commercially mature technology, are the most expensive part of them. Generally, precious metal NPs such as Pt, Ir, and Ru on carbon supports are used in FCs. However, the high cost of precious metals remains a main challenge for the large‐scale commercial application of FCs [271, 272, 273, 274]. Moreover, a major concern is finding an efficient, low‐cost, and stable catalyst for the sluggish ORR in the cathode part. To overcome the slow kinetics of the ORR, the cathode part requires high loading amounts of precious metal catalysts (generally Pt‐based catalysts) for surface reactions at a reasonable rate, which also increases the cost of the PEMFC technology. In addition, the inhomogeneous and wide‐size distribution of precious metal NPs on carbon support results in deteriorated stability and activity of the FCs. Recently, the ALD has been broadly used to precisely control the NP size, distribution, and catalyst‐support interaction on the support materials for the FC application [271, 272, 273, 274]. In this regard, our group reported several papers on precise designing and homogeneous distribution of catalyst NPs on the carbon support by the fluidized bed reactor atomic layer deposition (FBR‐ALD) for PEMFC application [54, 63, 275]. Besides the single‐metal NPs, the FBR‐ALD has also been used for the precise synthesis of bimetallic NPs like PtNi alloy on carbon support for enhanced FC ORR activity and stability [54, 63]. Moreover, interface engineering of catalysts through the self‐limiting surface reaction between the chemisorbed species on the support and injected precursors facilitates controlled growth and excellent uniformity of metal oxide films and metal NPs. In addition, the FBR ‐ALD reactor can be used to precisely decorate the catalyst NPs with TiO_2_ and SnO_2_ layers that are stable under acidic conditions to prevent dissolution and preserve the activity of the original catalyst NPs [54, 63, 275].

SOFCs have also emerged as promising for next‐generation FC devices that operate at low temperatures (<500°C) with minimum ohmic losses. However, the kinetics of sluggish ORR drops at low operational temperatures which reduces the overall electrochemical performance of the SOFC. Recent studies have shown that precise modification of surfaces of the stable metal oxides such as doped Al_2_O_3_, SnO_x_, TiO_x_, ZrO_2_, CeO_x_, and perovskite‐based materials (e.g. Pr_6_O_11_ and (Mn_0.8_Co_0.2_)_3_O_4_) and pure metals/alloys (e.g. Pt, Pd, Ru, Ni, Al, NiPd, PtRu, and NiRu) via the ALD technique significantly accelerated the SOFC performance. It is worth noting that ALD has been broadly applied for atomistic modification of the materials composition, morphology, and electrode‐electrolyte interface, to overcome critical issues such as durability of catalysts, low redox kinetics, and low ionic conductivity and over prolonged cycling activity in the FC technology [34, 276, 277, 278, 279, 280, 281, 282, 283, 284, 285, 286, 287, 288].

### 4.5 ALD in Battery Systems

**FIGURE 18:**
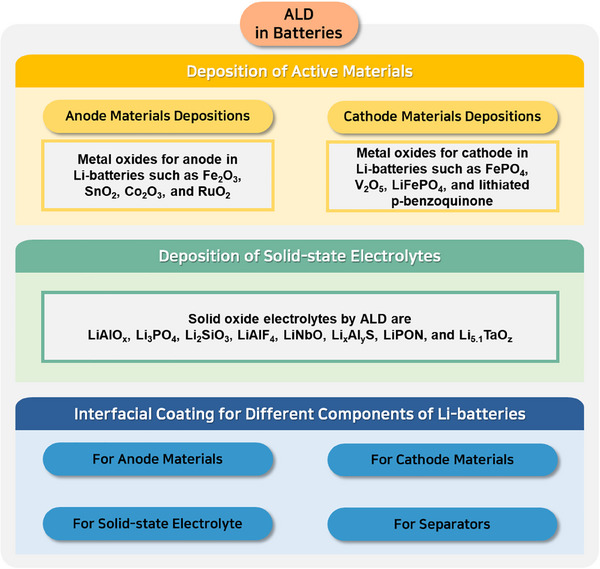
A flow chart diagram shows the utilization of ALD in the battery system.

A battery is an electrochemical device that converts the storable chemical energy in its active materials directly into electricity using electrochemical oxidation and reduction reactions. The batteries have drawn significant attention in modern technology due to their capability to store the electricity produced from renewable energy sources (e.g. solar and wind energies) as chemical energy. Typically, a unit cell of a battery contains an anode (negative electrode), and a cathode (positive electrode) stacked by a separator that is ionically conducting and electronically insulating in between them. We will emphasize the lithium (Li) ion batteries (LIBs) that are worldwide recognized in the modern technological era and also these batteries are largely utilized for domestic purposes, electric vehicles, and smart solar grids for electricity storage devices [289]. However, safety issues and the limited performance of the Li‐based batteries motivate the researchers to design novel materials and perform atomic scale engineering of the existing materials. Moreover, some well‐known challenges of the Li‐based batteries such as limited life cycle, low energy density, dendrite formation, structural instability, and volume expansion remain major concerns. The ALD technique has widely been used to overcome these issues by proper designing and atomic scale engineering of the active materials [289, 290]. Utilization of the ALD technique for various materials designing and interfacial engineering of electrodes and separators in the battery systems has been shown in Figure 18.

Various metal oxides as anode materials such as Fe_2_O_3_, RuO_2_, SnO_2_, and Co_3_O_4_ in Li‐batteries have been deposited by the ALD technique owing to their good charge storage efficiency [291, 292, 293, 294, 295]. For example, SnO_2_ thin film was deposited by ALD on a stainless‐steel (SS) current collector as anode material that exhibited higher properties for LIB with a stable battery efficiency of 646 mAh g^−1^ over 250 cycles. The experimental results showed that the ALD‐SnO_2_ thin film facilitates the Li‐ion deintercalation and also better charge storage capacity of the Li‐batteries was obtained by changing the phase structure (from amorphous to crystalline) of the ALD‐SnO_2_ thin films on SS. Many works have been reported on the deposition of ultra‐thin, conformal, and continuous anode materials on different three‐dimensional substrates (e.g. carbon nanotubes, Ni foam, and TiN nanotubes arrays for improving charge storage capacity and cyclic stability of Li‐batteries [291, 292, 293, 294, 295].

Also, Various cathode materials both lithiated such as LiCoO_2_ and Li_x_Mn_2_O_4_ and non‐lithiated like FePO_4_ and V_2_O_5_, in Li‐battery have been deposited by the ALD technique [296, 297, 298, 299]. For instance, conformal and homogeneous FePO_4_ as a cathode material was deposited on nitrogen‐doped carbon nanotubes (NCNTs) by taking advantage of the ALD technique by Liu et al. [300]. The fabricated FePO_4_/NCNTs showed a discharge current of 177 mAh g^−1^ at 1 coulomb (C) capacity and 141 mAh g^−1^ as a discharge capacity with the retention of 100% efficiency after 100 cycles. Moreover, Liu et al. [301] deposited a lithiated LiFePO_4_ cathode on carbon nanotubes (CNTs) substrates by ALD subcycles for Fe_2_O_3_, PO_x_, and Li_2_O. The fabricated LiFePO_4_/CNT cathode showed a good discharge current of 150 mAh g^−1^ at 0.1 C. Moreover, the cathode preserved a discharge current of 71 mAh g^−1^ at 60°C and maintained a charge capacity of 80% at 1 C after 2000 cycles.

Moreover, the ALD technique has attracted significant interest in the fabrication of conformal coating of proper materials for solid‐state electrolytes (SSEs) which are considered as the future high‐power density LIBs [302]. Compared to liquid electrolytes, the SSEs ensure safer use with excellent power density. Various materials such as LiAlO_x_, Li_3_PO_4_, Li_2_SiO_3_, LiNbO, LiAlF_4_, Li_x_Al_y_S, and Li_5.1_TaO_z_ have been deposited using ALD for SSEs [303].

Besides active materials deposition and SSEs fabrication, the ALD technique is renowned for interfacial coatings in battery systems to prevent the degradation of active materials during charging and discharging conditions. The ALD has been utilized for interfacial coating on the anode, cathode, SSEs, and separators in the battery systems. Precise control over interfacial coating thickness not only suppresses the degradation of active materials but also greatly improves the interfacial charge transfer kinetics and diminishes interfacial resistance [304, 305, 306, 307, 308, 309, 310, 311, 312, 313]. Some recently published ALD‐coating for interfacial engineering and atomistic reaction modulation in battery systems have been summarized in Table 10.

**TABLE 10:** Different ALD‐deposited interfacial metal oxide layers in battery systems.

Battery component	Interfacial coating	Battery performance	Ref.
Si NPs as anode material	ZnO layer ∼3 nm	1500 mAh g^−1^ over 260 cycles	[304]
Li anode in Li‐S battery	Al_2_O_3_ layer	90% retention for over 100 cycles	[305]
Na‐anode in Na‐metal battery	25‐cycle ALD‐Al_2_O_3_ coating	500 h long cycle life at 3 mA cm^−2^	[306]
Li_1.13_Mn_0.54_Ni_0.13_Co_0.14_O_2_ cathode in Li‐battery	ALD‐FeO_x_ coating	Battery capacity of 221 mAh g^−1^ at 1 C with ∼73% retention	[307]
Li_2_S impregnated in graphene foam as a cathode in Li‐battery	ALD‐Al_2_O_3_ coating ∼1 nm	The battery capacity of 866 mAh g^−1^ with 85% retention over 150 cycles	[308]
(Li_1.3_Al_0.3_Ti_1.7_(PO_4_)_3_) (LATP) as SSE	ALD‐coatings of Al_2_O_3_	A high ionic conductivity of 0.1 mS cm^−1^	[309]
Li_7_La_2.75_Ca_0.25_Zr_1.75_Nb_0.25_O_12_ (LLCZN) as SSE	ALD‐Al_2_O_3_ coating	A low interfacial impedance of 1 Ωcm^2^	[310]
A 3D porous garnet‐type SSE	ALD‐ZnO coatings ∼30 nm	Improves the Li wettability and reduces the interfacial impedance Li/SSE	[311]
Polyvinylidene fluoride‐hexafluoropropylene (PVDF‐HFP) as separator	ALD‐Al_2_O_3_ coating ∼30 nm	High thermal stability up to 200°C without shrinkage and fire‐resistant property	[312]
PVDF and commercial Celgard (PE/PP/PE) as separators	Al_2_O_3_‐coating by atmospheric ALD	Improves the electrolyte wettability and electrolyte uptake by 256%	[313]

### 4.6 ALD for Supercapacitors

Supercapacitors (SCs) have attracted considerable attention in the last few years due to their advantages of rapid charging, tolerance to a broad temperature range, eco‐friendliness, safety issues, and maintenance‐free operation over the batteries [314]. Typically, the SCs are electrical energy storage devices with fast charging‐discharging ability where electrode materials play a key role in attaining the final performance [314]. Generally, the SCs are made of metal oxides with high surface area, good electric conductivity, high stability, and low cost. However, most of the SCs suffer from low charging rates, self‐discharging, high current leakage, and poor cycling stability [315]. Recently, the precise deposition of electrode materials and their atomistic surface modification by the ALD technique facilitated overcoming these above issues. For example, an electrode with an ALD‐iron oxide (Fe_2_O_3_) layer on a nanostructured graphite foam carbon nanotube (GFCNT@Fe_2_O_3_) was reported by Guan et al. [316] The fabricated GFCNT@Fe_2_O_3_ electrode exhibited an ultrahigh energy density of 74.7 Wh kg^−1^ with a cycling ability of >50,000 cycles. Moreover, Hong et al. [317] reported an activated carbon electrode with an Al_2_O_3_ dielectric layer (2 nm thickness) by the ALD technique. The thin Al_2_O_3_ layer suppressed the electrolyte degradation and protected the surface functional groups of activated carbon, resulting in improved electrochemical performance of the electrode by 39% at 3 V. Furthermore, a core–shell type NiCo_2_O_4_/MoO_2_ electrode was coated with a uniform and thin outer layer of NiO by the ALD technique [318]. The NiO outer layer coated NiCo_2_O_4_/MoO_2_ electrode effectively reduced the capacity loss (<3%) after 20,000 cycles facilitated to achieve a power density of 136 Wh kg^−1^.

Besides metal oxide deposition, ALD has also been used for metal nitride and sulfide deposition for high‐performance SCs. For instance, Yang et al. [319] reported titanium nitride (TiN)‐based SCs with ultrafast charging ability and suppressed self‐discharging. The SC showed high energy and power densities with a minimal (< 1 µA) current leakage. In addition, TiN‐based ternary metal oxide (NiCo_2_O_4_@TiN) core–shell electrodes improved the performance of SCs [320]. In addition, the ALD technique has been used to fabricate MoS_2_ on 2‐dimensional (2D) stainless steel and 3‐dimensional (3D) Ni‐foam substrates by Kim group [321]. The MoS_2_@3D Ni‐foam as electrode showed an excellent capacitance of 3400 mF cm^−2^ with a 3 mA cm^−2^ current density and stability over 4500 cycles (with >80% retention). Furthermore, an ALD‐ZnS thin film was deposited precisely and uniformly on the ZnO nanowire arrays for the fabrication of the ZnO@ZnS core–shell electrode by the Li group [322]. The core–shell electrode was used as a binder‐free SC that exhibited outstanding performance.

### 4.7 ALD for Next‐Generation Batteries and Solar‐to‐Fuel Conversion Systems

Besides protective layer coating and surface/interface engineering of electrocatalysts and photoelectrocatalysts, ALD can be used in next‐generation batteries (e.g. Li‐S, Na‐ion, and multivalent batteries) and advanced solar‐to‐fuel conversion systems (e.g. integrated tandem PEC cell). ALD has been actively used in battery technologies for depositing ultra‐thin and conformal coatings. Moreover, it can effectively coat a thin and conformal protective layer at the electrode surface to greatly improve the stability of various electrodes for next generation batteries. For example, poor contact and high interfacial resistance between solid electrolytes and electrodes cause inferior performance in solid‐state batteries, however ultra‐thin and conformal interfacial layers by ALD can improve ionic conductivity and reduce interfacial resistance [289, 290]. Moreover, in Li‐S batteries, polysulfide shuttle effect causes capacity fading that can be overcome by encapsulating sulfur cathodes with ALD‐grown ultra‐thin TiO_2_ or Al_2_O_3_ layers to prevent polysulfides transfer and enhance cycle life. ALD can also be used in developing advanced anode materials by coating them with stabilizing layers to prevent electrolyte side reactions and mechanical failure for improving poor cycling‐capacity and stability of Na‐ion and multivalent batteries.

On the other hand, ALD offers solutions for many of the challenges in solar‐to‐fuel conversion systems, such as PEC water‐splitting and CO_2_ reduction that require high‐performance and durable photoelectrodes. Photoelectrode materials (e.g. Si, GaAs, perovskites, and metal oxide semiconductors) are prone to corrosion under operational conditions. Therefore, depositing ultra‐thin and conformal oxide layers (e.g. TiO_2_, SnO_2_, and Al_2_O_3_) over photoelectrode materials can effectively protect them from photocorrosion without impeding light absorption or charge transfer properties [225, 235, 236]. ALD can also be utilized for proper surface and interface engineering of photoelectrodes to improve poor catalytic efficiency and slow reaction kinetics of water‐splitting and CO_2_ reduction reactions. It is worth mentioning that ALD offers high‐performance catalysts (e.g. NiFe oxides, RuO_2_) synthesis with atomic‐scale precision to optimize active sites. The most challenging issue in solar‐to‐fuel conversion systems is poor charge transfer between light absorbers, catalysts, and the electrolyte. This issue can be greatly overcome by engineering interfaces with ALD to enhance charge transport and minimize recombination losses in photoelectrodes. ALD‐grown layers can bridge the electronic gap between a semiconductor photoelectrode and an electrocatalyst. It is noted that single‐layer coatings may not maintain balance protection, activity, and charge transfer properties. Therefore, deposition of hybrid and multi‐functional layers (e.g. conductive oxides over corrosion‐resistant layers) would facilitate in improving performance of photoelectrodes. Importantly, it is required to scaling up solar‐to‐fuel conversion systems while ensuring stability of photoelectrodes in outdoor conditions. Scalable ALD techniques can be used to protect large‐area photoelectrodes and outdoor PEC systems for practical application.

Beside unique advantages of ALD, it has some limitations that need to be addressed for its widespread applications. One significant drawback of the ALD technique is its relatively low deposition rate compared to other vapor deposition techniques such as PVD and CVD owing to its layer‐by‐layer growth mechanism based on self‐limited reactions. This inherently slow process requires longer deposition times, particularly for thicker films. The deposition rate is influenced by factors such as deposition temperature, the choice of precursors and reactants, and type of substrate and its surface chemistry. To overcome this drawback, more advanced variants of ALD technique such as PEALD, EA‐ALD, batch‐ALD, SALD, FBR‐ALD, and MLD have been developed [23, 323]. In ALD technique, a substantial fraction of the precursor often estimated at approximately 60% remains unreacted, leading to considerable resource wastage, energy consumption, and increased labor costs. Additionally, most of the ALD precursors are expensive, this inefficiency poses a fundamental economic limitation. Therefore, optimizing precursor utilization through appropriate process design could significantly reduce operational costs and promote the wider adoption of ALD in industrial applications [26]. It is worth mentioning that utilization of ALD on high‐surface‐area substrates such as nanopowders and other 3D nanostructures facilitates better precursor utilization compared to planar substrates. Moreover, active development of novel ALD‐precursors and reactants is essential to enable the deposition of high‐quality materials through optimized reaction pathways for diverse applications.

## 5 Summary and Outlook

ALD has emerged as a promising and advanced technology for depositing diverse materials with conformal and precise control of thickness at atomic scale. In this review, we comprehensively address the recent advancement of catalysts and their atomic‐scale reaction modulation by the ALD technique for energy conversion, generation, and storage applications, particularly emphasizing electrochemical systems such as electrocatalysis and photoelectrocatalysis for hydrogen fuel generation from water‐splitting reactions, solar cells, fuel cells, batteries, and supercapacitors. The important role of the ALD in controlling the size of catalysts and solving their agglomeration issue has been elaborated. Moreover, we demonstrate the capability of the ALD technique for efficient catalyst design by citing recently reported works, in particular, the emphasis has been given to the surface/interface engineering of catalysts for efficient charge carrier transport, surface passivation, prevention for catalyst dissolution, tuning the interfacial (electrode‐electrolyte) reaction kinetics in energy conversion and storage applications. More research on ALD‐based routes for catalyst synthesis and atomistic reaction modulation to get insight into the reaction mechanism may revolutionize the field of sustainable energy conversion and storage.

The above discussion represents that ALD is an emerging technique for energy generation, conversion, and storage applications. However, some important issues of ALD technology must be addressed to utilize this technology in practical energy research. For instance, firstly, the cost of the ALD technology must be reduced for particular energy‐related applications. Generally, ALD is a time‐consuming, sophisticated, high‐cost technique due to its slow materials deposition rate, upfront equipment cost, maintenance, throughput, and limited precursors. The cost analysis and sustainability of the ALD technique should be exclusively explored. Moreover, the conditions or parameters of the ALD technique such as deposition temperature, chamber pressure, and the chemistry of precursor and reactant vividly affect the quality, conformality, crystallinity, and interfacial properties of thin films. Therefore, it is of utmost importance to accurately explore the surface reactions between ALD‐coated materials and the underlying substrate for precise control of the coated film and the interfacial properties. To gain insight into the materials growth mechanism in ALD, in‐situ or Operando studies of the ALD technique should be performed.

On the other hand, synthesizing supported NPs with controlled shape and size and their applications in catalysis have attracted considerable attention owing to their unique catalytic properties. By taking advantage of advanced variants of ALD (e.g. thermal‐ALD, PEALD, EA‐ALD, batch‐ALD, SALD, FBR‐ALD, and MLD), it is possible to synthesize the catalysts at the atomic scale, particularly, site‐selective ALD provides tremendous opportunities in catalytic efficiency and selectivity studies. The recent interest in atomic‐scale precision of catalysts is proof of the importance of this branch of ALD. More future works on the advanced ALD technique would be helpful in the synthesis of novel catalysts and selective ALD techniques for energy applications. Of course, the ALD technique could be developed for precise synthesis of catalysts by considering cost minimization and related future work would be focused on precious metal‐free catalysts.

## Author Contributions

Myung‐Jin Jung and Alireza Razazzadeh contributed equally to this work on writing, literature survey, and schematic drawing. Hasmat Khan and Se‐Hun Kwon conceptualized, wrote, edited, and supervised the manuscript.

## Conflicts of Interest

The authors declare no conflicts of interest.
